# The Asian Pacific Association for the Study of the Liver clinical practice guidance: the diagnosis and management of patients with autoimmune hepatitis

**DOI:** 10.1007/s12072-021-10170-1

**Published:** 2021-05-04

**Authors:** Guiqiang Wang, Atsushi Tanaka, Hong Zhao, Jidong Jia, Xiong Ma, Kenichi Harada, Fu-Sheng Wang, Lai Wei, Qixia Wang, Ying Sun, Yuan Hong, Huiying Rao, Cumali Efe, George Lau, Diana Payawal, Rino Gani, Keith Lindor, Wasim Jafri, Masao Omata, Shiv Kumar Sarin

**Affiliations:** 1 Peking University First Hospital, Beijing, China; 2 Teikyo University School of Medicine, Tokyo, Japan; 3 Beijing Friendship Hospital, Capital Medical University, Beijing, China; 4 Shanghai Institute of Digestive Disease, Renji Hospital, School of Medicine, Shanghai Jiao Tong University, Shanghai, China; 5 Department of Human Pathology, Kanazawa University Graduate School of Medicine Kanazawa, Kanazawa, Japan; 6 Fifth Medical Center, Chinese PLA General Hospital, Beijing, China; 7 Beijing Tsinghua Changgung Hospital, Beijing, China; 8 Peking University People’s Hospital, Beijing, China; 9 Department of Gastroenterology, Harran University, Şanlıurfa, Turkey; 10 Humanity and Health Medical Group, Hong Kong Special Administrative Region, China; 11 Department of Hepatology, Cardinal Santos Medical Center, Manila, Philippines; 12 Department of Internal Medicine, Cipto Mangunkusumo Hospital, University of Indonesia, Jakarta, Indonesia; 13 College of Health Solutions, Arizona State University, Phoenix, AZ USA; 14 Aga Khan University, Karachi, Pakistan; 15 Department of Gastroenterology, Yamanashi Prefectural Central Hospital, Kofu-City, Yamanashi Japan; 16 The University of Tokyo, Tokyo, Japan; 17 Institute of Liver and Biliary Sciences, New Delhi, India; 18 Peking University International Hospital, Beijing, China

## Introduction

Autoimmune hepatitis (AIH) is not a common disease but with increasing incidence in Asia–Pacific area. Although the etiology and pathogenesis are not completely clear, the disease is the result of loss of autoimmune tolerance in genetically predisposed individuals. AIH is characterized by autoantibodies, hypergammaglobulinemia, and interface hepatitis. Corticosteroids and azathioprine are standard regimens for AIH, which should be started after diagnosis. Alternative therapy should be considered for those who do not respond to standard regimens. End-stage AIH patient could be saved by liver transplantation.

A panel of clinicians discussed the epidemiology, pathology, diverse clinical characteristics and therapy of patients with AIH in the Asia–Pacific region and wrote the draft of this guidance. Particular attention is paid to those characteristics that are different from those of Western patients. Internal discussion and external review were performed before finalizing this guidance.

## Epidemiology

AIH is a rare disease with unknown etiology and may affect all ages, genders and ethnic groups worldwide. However, this disease presents with a strong female predominance and geographic variation [1]. As summarized in a recent meta-analysis, the AIH has a global annual incidence of 1.37 per 100,000 (1.11 for female and 0.22 for male) and prevalence 17.44 per 100,000 (12.77 for female and 2.91 for male) [2]. The pooled annual incidence rate in Asian (1.31/100,000) is similar to that in European (1.37/100,000) and American population (1.00/100,000); however, the pooled prevalence rate in Asian (12.99/100,000) is lower than that in European (19.44/100,000) and American population (22.80/100,000) [2]. One possible explanation for this incidence–prevalence discrepancy would be the late onset or diagnosis and shorter survival of the AIH in Asian population [3, 4].

The exceptional case in Asia is Japan, where the point prevalence of AIH in Japan was reported to be 23.90/100,000, comparable to those in Europe and America [5], while a recent study in Japan suggests a peak age of AIH patients in Japan is 60–70 years [5]. Indeed, even within Asia, the reported prevalence of AIH varies greatly. In Korean, a population-based study using the database of the National Health Insurance (which covers 95% of the South Korean population) demonstrates a low average annual incidence and prevalence rate with 1.07 and 4.82 per 100,000 people, respectively [6].

Disparities in prevalence of AIH in different geographical regions may have several explanations. First, genetic factors may play an important role and this can partly explain the higher prevalence rate of AIH in New Zealand than other Asia–Pacific countries, as 83% of the population is of European descent [7]. Second, environmental factors may also play a role in the development of AIH, as supported by a recent study showing biological use was associated with increased incidence of AIH in Iceland [8]. Third, health care system difference and relatively low awareness/recognition of AIH in Asia [4]. Fourth, the methodological differences in term of study design, population selection and database utility, case definition, case finding and ascertainment [2].

## Pathogenesis

AIH is caused by a lack of autoimmune tolerance, and the etiology and pathogenesis are not completely clear. With the deepening of AIH-related mechanism research, the pathogenesis of this disease has made some progress. AIH is the result of interactions between genetic susceptibility, predisposing factors, molecular mimicry, autoantigen response, and immunomodulatory defects.

### Genetic susceptibility

The etiology of AIH remains elusive, however, both genetic and environmental factors are thought to play a crucial role in the development of the disease. Although there have been no family studies in AIH, many case reports revealed that AIH occurred simultaneously in two or three family members [9]. Epidemiological studies also found a family history of autoimmunity in about 40% of AIH patients, as well as the significantly increased percentage of AIH patients concurrent with extrahepatic autoimmune disorders. These data indicated an important role for a genetic component in the development of AIH [10]. Till now, genetic studies have identified multiple loci influencing the susceptibility to AIH in human leukocyte antigen (HLA) and non-HLA regions. Despite the ethnic differences, HLA-DR3 or -DR4, is the most convincing genetic risk loci for AIH (Table 1) [11, 12]. HLA-DRB1*04:04 and DRB1*04:05 were considered to increase the susceptibility of AIH in the Mexican, Korean, and Japanese populations, while HLA-DRB1*03:01 and DRB1*04:01 have been described as the most significant disease susceptible loci that related to the prognosis of AIH among European and American populations [13]. In contrast, some alleles like DRB1*1501 and DRB1*1302 were demonstrated as protective factors in AIH [14, 15]. HLA B27 and Cw4 had significant association with AIH-1 in Western Indian population [16], while HLA-DRB1*14 indicated high risk of AIH-2 in North India [17]. In addition, more and more non-HLA genes were reported to participate in AIH, such as tumor necrosis factor-receptor superfamily member 6 (TNFRSF6), protein tyrosine phosphatase N22 (PTPN22), vitamin D receptor (VDR) and so on [18–20]. The only genome-wide association study of AIH to date was performed in Caucasians, which suggested association between AIH and variants mapping to Scr homology 2 adaptor protein 3 (SH2B3) and caspase recruitment domain family member 10 (CARD10) gene [21]. In addition, candidate gene association studies have been conducted in AIH, but the results were not consistent across different populations [13, 21, 22].

**Table 1:** HLA alleles associated with AIH

Year	Author	Populations	Type of AIH	Risk loci	Protective loci
1992	Seki et al. [23]	Japanese	–	DRB1*04:05	–
1997	Strettell et al. [24]	White and of northern European	1	DRB1*03:01, DRB1*04:01	DRB5*01:01, DRB1*15:01
1997	Czaja et al. [25]	–	1	DRB1*03:01, DRB1*04:01	–
1998	Vazquez-Garcia et al. [26]	Mexican (Mestizo)	1	DRB1*04:04	–
1999	Bittencourt [27]	White, black and Amerindian ancestry	1 and 2	DRB1*03, DRB1*13, DRB1*07	DRB1*03:01
2003	Amarapurkar [16]	Western Indian	1	B27, Cw4	–
2005	Muratori et al. [28]	Italian and Caucasian	1 and 2	B8-DR3-DQ2	–
2006	Teufel et al. [29]	German	1	B8-DR3-DQ2	–
2006	Al-Chalabi et al. [30]	British	1	B8-DR3/DR4	–
2006	Djilali-Saiah et al. [31]	Caucasian	2	DQB1 *02:01,	–
2007	Mdel et al. [32]	Mestizo	1	DRB1*03:01, DRB1*13:01	DQB1*04
2008	Lim et al. [33]	Korean	1	DRB1*04:05, DQB1*04:01	
2014	de Boer et al. [21]	Netherlander, German, and Switzer	1	DRB1*03:01, DRB1*04:01	–
2014	Umemura et al. [14]	Japanese	1	DRB1*04:05, DQB1*04:01	DRB1*15:01, DQB2*06:02
2014	Kaur [17]	North Indian	2	DRB1*14	–
2017	Oka et al. [15]	Japanese	1	DRB1*04:01, DRB1*04:05, DQB1*04:01	DRB1*13:02

AIH is classified into two types (AIH-1 and AIH-2) according to different autoantibodies, which are described in detail in “Laboratory findings”

### Potential predisposing factors and destruction of autoimmune tolerance

Environmental factors have also been linked to the pathogenesis of AIH. Exposure to drugs like minocycline and nitrofurantoin, low vitamin D levels and pathogens were known risk factors for AIH [34–37]. Molecular mimicry hypothesis was developed to explain the production of autoantibodies in autoimmune disorders. Exogenous pathogens, such as bacteria or virus, may trigger the production of antibodies and activate immune response [38]. The main target cell of the disease process is the hepatocyte. Certain drugs, including nitrofurantoin, minocycline, oxyphenisatin, methyldopa, diclofenac, interferon *α*, pemoline, atorvastatin, infliximab and herbal agents, can induce hepatocellular injury that imitate AIH [39]. The frequency of drug-induced AIH among patients with classical AIH is 9%, and most cases present acute onset [40]. Classical AIH is typically continuous after the incriminated agent removal [41], which implies the triggering antigen is constantly renewed or immune regulatory mechanism is permanently impaired [41]. In particular, re-exposure to the offending agent should be avoided indefinitely after recovery. More recently, intestinal microbiota has been suggested to be involved in all types of AIH [42]. For example, alterations in intestinal microbiota were found in AIH, and translocation of a gut microbe to the liver promoted autoimmunity in patients [43, 44]. The enrichment of *Veillonella dispar* in the steroid-naïve AIH microbiome was correlated with disease severity. Intestinal microbiota may be a useful non-invasive biomarker in all types of autoimmune hepatitis [45]. 

**Fig.1:**
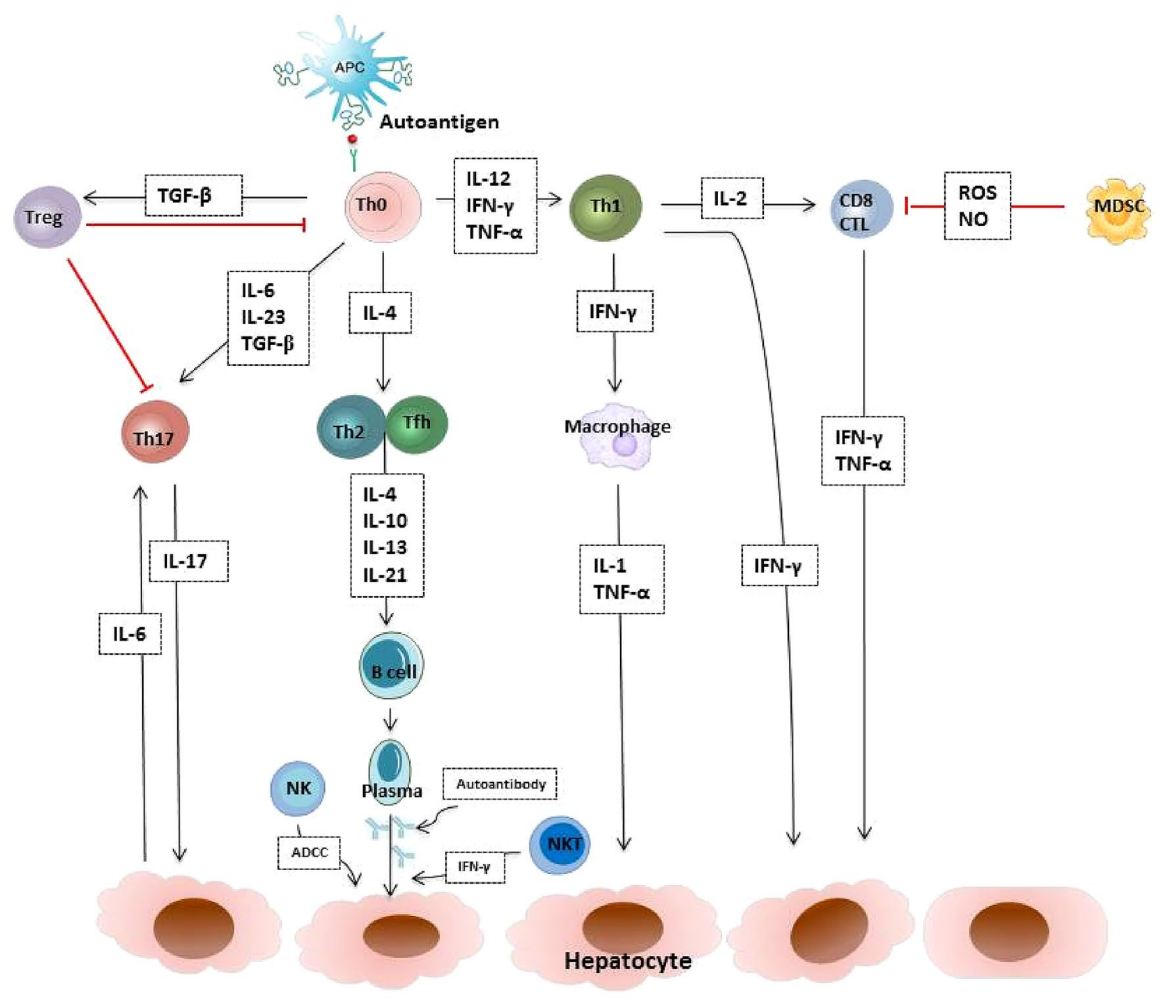
AIH causes a cycle of immune injury to hepatocytes. The immune imbalance between effector T cells, regulatory T cells, B cells, NK cells and MDSCs is a critical reason for autoimmune-mediated liver damage. *APC* antigen presenting cell, *Th* T helper cell, *CTL* cytotoxic T cell, *Treg* regulatory T cells, *Tfh* T follicular helper cell, *MDSC* myeloid-derived suppressor cell, *NK cell* natural killer cell, *ADCC* antibody-dependent cellular cytotoxicity, *TGF-β* transforming growth factor-β, *IFN-γ* interferon-γ, *TNF-α* tumour necrosis factor-α, *IL* interleukin

The hypotheses on the pathogenesis of AIH are components of the humoral and cellular immune system. There is a complex nature of immune cells, signaling pathways and their interactions in AIH, including regulatory T cells (Treg cells) loss of self-tolerance, accelerated effector T cells activation, increased autoreactive B cells production and natural killer (NK) cells activity (Fig. 1). Autoantibodies may be useful in identifying targets of disease processes ultimately mediated by T cells. Numerical and functional Treg defects were observed in AIH patients [46–49]. The transfusion of ex vivo expanded Treg cells might be a potential curative approach for AIH. The serum levels of IL-17 and IL-23, as well as the frequency of IL-17^+^ cells in the liver, were significantly elevated in patients with AIH and associated with increased inflammation and fibrosis. Th17 cells are key effector T cells that regulate the pathogenesis of AIH, via induction of MAPK dependent hepatic IL-6 expression. Blocking the signaling pathway and interrupting the positive feedback loop are potential therapeutic targets for AIH [50]. Recently, myeloid-derived suppressor cells (MDSCs), a heterogeneous immature myeloid cell population with remarkable immune suppressor function as an important negative feedback mechanism in immune-mediated liver injury. MDSCs have a remarkable ability to suppress T cell responses and regulate innate immunity by modulating cytokine production and interacting with natural killer cells. In autoimmune liver disease, Farnesoid-X-receptor (FXR) activation expanded monocytic MDSCs in liver. FXR activation upregulate the expression of PIR-B by binding PIR-B promoter to enhance the suppressor function of MDSCs [51]. Maintaining the balance between Treg cells, Th17 cells, MDSCs and NKT cells has become the promising strategy of future immune therapy.

### Clinical spectrum

AIH has changeable clinical phenotypes and different presentations [11]. The spectrum of initial manifestations ranges from asymptomatic to acute hepatic failure, but the major manifestation is chronic hepatitis [52, 53]. The clinical manifestations of AIH are non-specific [54], around 25% AIH patients are asymptomatic. Asymptomatic patients are generally discovered because of abnormal liver function tests in physical exam. The most common symptoms are sleepiness, fatigue, and general malaise. Hepatomegaly, splenomegaly, ascites and occasional peripheral edema can be observed during physical examination. Because of the insidious onset, nearly 30% of AIH patients demonstrated histological cirrhosis at accession [4, 55]. In some cases, decompensated manifestations could be the initial presentations. The characteristic biochemical features of AIH are the elevated AST and ALT levels, indicating hepatocellular injury. About 10–20% of the patients are asymptomatic and only have elevated serum aminotransferase levels during the physical examination. The risk of progression to cirrhosis in these patients is similar to that in the symptomatic patients. The first onset of AIH can occur in pregnant female or after their childbirth. Therefore, early diagnosis and timely treatment are vital for the safety of both mothers and infants [56].

AIH is usually complicated with autoimmune diseases including Hashimoto’s thyroiditis (10–23%), diabetes mellitus (7–9%), inflammatory bowel disease (2–8%), rheumatoid arthritis (2–5%), Sjögren’s syndrome (1–4%), celiac disease (%3.5) psoriasis (3%) and systemic lupus erythematosus (SLE) (1–2%), and so on [57]. AIH and other autoimmune diseases such as SLE are all independent disease types. Simultaneously present autoimmune diseases shall be treated according to the major disease type, and the dosage of glucocorticoids should be exactly sufficient to control disease activities.

### Acute and acute severe AIH

AIH generally has a chronic presentation with serum ALT and/or AST abnormalities, whereas it can also present as an acute/acute severe disease [58]. The clinical features of acute AIH from the Japanese population mainly include elevated ALT and a higher proportion of liver fibrosis [59]. A Japanese nationwide survey found about 10% of AIH cases showed acute hepatitis in histological examinations [60]. Zone 3 necrosis is a histological feature of acute AIH [61]. Recently, the Asian Pacific Association for the Study of the Liver (APASL) acute on chronic liver failure (ACLF) working party has reported that AIH flare as a cause of ACLF is not uncommon in Asian patients (82/2825 ACLF patients, 2.9%) [62]. When AIH presents acute/acute severe onset, patients may not have high immunoglobulin G (IgG) concentrations and serum autoantibodies to fulfill the IAIHG diagnosis criteria, especially using the simplified criteria [61, 63, 64]. Furthermore, histological features may also be atypical, these AIH patients are likely to be misdiagnosed as cryptogenic hepatitis. Patients with advanced encephalopathy should be considered for liver transplantation promptly [65].

### AIH patients with cirrhosis or at liver decompensated stage

About 1/4 of AIH patients are cirrhotic at presentation [4, 66]. Miyake et al. established a model for determining cirrhosis in type I AIH patients. The risk score (≥ 0.20) was estimated to be cirrhotic and the specificity and sensitivity was 83 and 90%, respectively [67]. From 1975 to 2010, Abe et al. found 20.4% of patients with type I AIH (*n* = 250) were cirrhotic in Japanese population. During the follow-up period, the relapse rate was high in patients who developed cirrhosis [68].

Hepatocellular carcinoma development in AIH does exist and is associated with cirrhosis, although it is less common than other causes of liver diseases [69]. For the cirrhosis subgroup, routine imaging examinations like ultrasound should be done at 6-month intervals to exclude hepatocellular cancer (HCC) [70].

### Recommendation

The major manifestation of AIH is chronic hepatitis. It can also present as acute attacks or even acute liver failure. Therefore, AIH should be considered for patients with abnormal liver function of unknown causes.

## Pathology

Liver histological examination is essential for the diagnosis and management of AIH. Liver biopsy is prerequisite for the accurate diagnosis of AIH [71–73]. It is also helpful to identify overlap syndromes [overlap of AIH with other diseases, such as primary biliary cholangitis (PBC), primary sclerosing cholangitis (PSC)], highlight the posible concomitant disease, and exclude other potential cause of liver diseases, such as drug-induced liver injury (DILI). Besides diagnosis, biopsy examination is used to assess fibrosis stage and grade disease severity in AIH. The four-tier grading systems developed for chronic viral hepatitis histological grading and staging systems, such as Metavir [74], can also be used to assess the grading of inflammatory activity and staging of fibrosis in AIH. These factors provide important information on prognosis and may serve to guide treatment decision [75–81]. Furthermore, histological evaluation helps to determine the completeness of the treatment response and appropriate timing for drug withdrawal, because the degrees of necroinflammatory activity and severity of AIH do not often correlate well with transaminase levels [79]. Liver biopsy at diagnosis, therefore, is recommended in all patients with suspected AIH unless there are significant contraindications, and it should be performed prior to stating treatment [12, 39, 66, 72, 73, 79, 82]. The transjugular approach can be used in cases with severe coagulopathy, particularly, in those with acute/fulminant onset of the disease [83–88]. Alternatively, biopsy under mini-laparoscopy can be used and has also been shown to be safe even in cases of advanced coagulopathy [89–91].

In certain circumstance, such as patients with contraindications of liver biopsy, or no transjugular liver biopsy being carried out, non-invasive methods to evaluate stage may be candidates. Growing interest for non-invasive methods for the assessment of fibrosis and inflammation in AIH [92–98]. But at present, non-invasive methods cannot substitute the need of a biopsy, particularly at diagnosis.

### Classical histologic features

Interface hepatitis (hepatitis at the junction of the portal tract and hepatic parenchyma, formerly called piecemeal necrosis) with dense lymphocytic or lymphoplasmacytic infiltrates, hepatocellular rosette formation, emperipolesis are representative findings of AIH [71–73, 99, 100]. Plasma cells are typical for AIH and usually found abundant at the interface and throughout the lobule [101–105], but their paucity in the inflammatory infiltrate (34% of cases) does not preclude the diagnosis [82, 99, 106–108]. However, there is no histopathological feature that is pathognomonic of AIH. Other individual histological features such as interface hepatitis, plasma cells, and emperipolesis are also not specific for AIH, and patients with other disease such as DILI, viral or immune-mediated diseases could show similar features [99, 109–119].

- Hepatic rosette: a small group of hepatocytes arranged around a small central lumen formation of the hepatocytes forming a pseudoglanular structure [82, 101]
- Emperipolesis has been widely described in classical AIH and included in the simplified International Autoimmune Hepatitis Group scoring system (2008) [73, 120]. The presence of emperipolesis indicates a close immune interaction between lymphocytes and hepatocytes, which may subsequently induce hepatocyte apoptosis and has been proposed as an additional mechanism of autoimmune-mediated liver injury in AIH [121].
- Other features of classical AIH
  - Giant cell hepatitis
  - Pigmented macrophage, Kupffer cell hyperplasia
  - Hyaline droplets in Kupffer cells, 50–74% in AIH [122–124], new proposed diagnostic hallmark
  - Bile duct injury usually mild–moderate (nondestructive cholangitis)
  - Endotheliitis, endothelial injury, sinusoidal infiltration
  - Granuloma

### Histologic features of acute presentation

Centrilobular necrosis (CN) is found in 21.8–100% of AIH patients with acute presentation [61, 101–103, 105, 125–127]. CN has been more frequently found in the acute hepatitis phase (100%) than in the acute exacerbation phase of AIH (42%) [61]. Its prevalence also increases with disease severity [128]. It was more often observed in AIH with an acute presentation than in that with a chronic presentation (53 vs. 17.5–30.7%) [61, 105, 129–131]. Some cases with CN and without or with minimal portal involvement, representing typical acute hepatitis, may progress to classic AIH [127, 132–136]. CN with plasma cell infiltration is useful for supporting the diagnosis of AIH with acute presentations.

Histological features of acute hepatitis, including CN, submassive necrosis and massive necrosis, were present in 12.2–88% cases [58, 61, 102, 127, 128, 137–143].

Other features in AIH with acute presentation:

• Interface hepatitis (66.7–92.3%) [63, 102, 104, 105, 127, 144]
• Hepatic rosette formation (14.6–60.5%) [61, 63, 105, 127, 137, 142, 145]
• Bile duct injury (21.1–77.8%) [63, 105]
• Fibrosis (40–96.1%) [61, 102–105, 126, 141, 144]
• End-stage fibrosis (stage 4) (0–33.3%) [58, 104, 126, 141, 144, 146].
• Portal inflammation (90–100%) [105, 126, 127]
• Lobular necrosis/inflammation (73–100%) [103–105, 126, 142, 144]
• Cobblestone appearance of hepatocytes (44.4–82.6%) [101, 142]

### Recommendations

Liver biopsy is very important for the diagnosis of AIH, but because there is no specific histological hallmark, an experienced pathologist is needed for the diagnosis. It is recommended to evaluate the degree of inflammatory activity by HAI score. For the difficult cases, communication with the clinicians is necessary.

## Diagnosis

The diagnosis of AIH is based on the presence of typical clinical, laboratory features (including increased serum immunoglobulin G (IgG)/gamma-globulin levels, presence of autoantibodies), combined with pathological examination, and exclusion of other causes of liver diseases [e.g., chronic viral hepatitis, alcoholic liver disease, non-alcoholic steatohepatitis (NASH), drug-induced liver injury, and Wilson’s disease].

### Clinical features

AIH primarily affects females, especially in childhood/teenage years and in middle age [66, 147]. Most of AIH patients has no apparent symptom or only present some non-specific symptoms. The presentation of AIH at onset is variable, ranging from asymptomatic to acute/severe or even fulminant, about one-fourth of AIH patients present with an acute onset, among these patients, some present with the acute exacerbation of chronic AIH, and others present with the true acute AIH without histological findings of chronic liver disease. One-third of patients at diagnosis have already developed cirrhosis due to delay in diagnosis, irrespective of the presence of symptoms.

### Laboratory findings

Increased immunoglobulin G/gamma-globulin levels, presence of circulating autoantibodies, and elevation of aminotransferase levels, are the important laboratory characteristics of AIH. Than et al. reported the level of IgG in Singapore Asian and United Kingdom Asian were 16.7–33.3 g/L and 15.5–30.6 g/L, respectively [148]. In Japan, the peak of serum IgG levels in AIH patients ranged from 15 to 20 g/L [149]. In China, Ma’s research group reported the mean IgG levels in classical AIH patients with decompensated cirrhosis were 24.1 g/L ranged from 10.9 to 48.4 g/L, but in the patients with autoantibody-negative AIH, the IgG levels were lower than those in the classical AIH [151, 152]. In serum biochemistry, the AIH patients have a typical hepatitic pattern, included elevation of serum aminotransferases or bilirubin, and normal or only mildly raised serum alkaline phosphatase. Serology is very important to the both diagnosis and classification of AIH, serological tests should be completed in all patients for the definitive diagnosis of AIH. Most of AIH present with one or more significant titers autoantibodies. ANA, smooth muscle antibodies (SMA) and antibodies to kidney microsome-1 (anti-LKM1) are standard autoantibodies. According to the pattern of autoantibodies detected, AIH is classified into two types, ANA and/or SMA antibody characterize type 1 AIH (AIH-1) which accounts almost for 90% of cases, anti-LKM1 and antibodies to liver cytosol type1 (anti-LC1), characterize type 2 AIH (AIH-2). However, these auto-antibodies are not fully AIH specific, as they may also be found in various liver disorders. Although antibodies to soluble liver antigen/liver-pancreas (anti-SLA/LP) are highly specific markers for AIH in both AIH-1 and AIH-2 [153, 154], none was positive for anti-SLA in 154 Japanese patients with type 1 AIH, anti-SLA/LP positivity seems to be unusual in APASL group [155]. The presence of an autoantibody is a common feature of AIH, in acute-onset AIH or corticosteroid-treated AIH, autoantibodies can be absent or loss [156, 157]. It was reported that in Japan, ANA negative or less than 1:40 were seen in 29% of severe or fulminant AIH, and 39% of acute-onset AIH patients [102, 142]. In China, circulating autoantibodies were absent in about 10.2% of AIH patients [152]. The nonstandard autoantibodies, including antibodies to actin (anti-Actin), antibodies to alpha-Actinin (anti-α-Actinin), atypical perinuclear antineutrophil cytoplasmic antibodies (p-ANCA) and antibodies to asialoglycoprotein receptor (anti-ASGPR), can support or extend the diagnosis of AIH whom the standard biomarkers are insufficient to render a diagnosis. Anti-SLA/LP characterizes type 1 AIH, and anti-LC1 characterizes type 2 AIH.

### Histology

Liver histology is important not only in confirming the clinical diagnosis of AIH, but also in differential diagnosis of AIH, it plays a major role in clinical diagnosis scoring systems. The typical histological features of AIH are precisely described in the pathology section of this guidance.

### Non-invasive assessment of fibrosis

Although liver biopsy is a golden standard for evaluation of the liver inflammation and fibrosis, it is an invasive and expensive procedure, non-invasive assessment methods are repeatable, inexpensive and well accepted. Several non-invasive laboratories and radiology-based methods have been developed to assess the stage of fibrosis in chronic liver diseases, including AIH.

The FibroTest^®^ [158], the serum AST/platelet ratio index (APRI) [159], the Fibrosis-4 index (FIB-4) [160], and the enhanced liver fibrosis (ELF) test [161], angiotensin-converting enzyme levels [162], neutrophil lymphocyte ratio, mean platelet volume, red cell distribution width [163–165] are possible candidate laboratory markers of hepatic fibrosis in AIH.

Transient elastography (FibroScan) is gradually replacing the liver biopsy as a reliable tool to monitor chronic liver disease, including AIH. In Asia–Pacific region, transient elastography was helpful in predicting significant liver fibrosis in AIH [166, 167].Chinese researchers reported the diagnostic accuracy of FibroScan for detecting of fibrosis in AIH patients. Liver stiffness measurement by FibroScan was superior to other non-invasive markers in assessing the fibrosis of AIH patients, and the optimal cut-off values of liver stiffness measurements was 6.27, 8.18 and 12.67 kPa for stage F2, stage F3 and stage F4, respectively [166]. Transient elastography can be routinely used for noninvasive staging of hepatic fibrosis in AIH patient either in the diagnosis at onset or during the follow-up. Acoustic Radiation Force Impulse (ARFI) elastography could differentiate significant from non-significant liver fibrosis in patients with AIH and this non-invasive method can also be used for monitoring fibrosis progression in AIH [168, 169]

A recent study has evaluated performance of magnetic resonance elastography (MRE) and findings of MRE were strongly correlated with advanced fibrosis stage in AIH [170]. The role of these non-invasive methods is a merit in assessing or monitoring the hepatic fibrosis, treatment response as well as disease outcome.

But hepatic inflammation, necrosis, and swelling can impact liver stiffness, such as excessive hepatocyte apoptosis and necrosis can activate HSCs, and the ongoing fibrogenesis can cause liver stiffness increased, in the concomitance of aminotransferase flares, especially in advanced fibrosis and cirrhotic patients presenting with a clinical pattern of acute hepatitis, non-invasive assessment methods are not reliable instruments.

### Diagnostic methods

#### Revised original diagnostic scoring system of the International Autoimmune Hepatitis Group (IAIHG) [72]

The revised original scoring system is a diagnostic method to ensure the systematic evaluation of patients [171]. This scoring system was based on 12 clinical components, originally developed as a tool for scientific purposes [172] (Table 2). It can distinguish AIH patients from cryptogenic hepatitis, and for patients suspected of AIH, the revised original Diagnostic scoring system can support the diagnosis by rendering a composite score of corticosteroid treatment response. In Asia–Pacific area, it was reported a sensitivity of 100% and a specificity of 93% to detect in Japanese AIH patients [173]. Though the revised original diagnostic criteria were incorporated into clinical diagnosis of AIH, it is a very complex score system, and even including a variety of parameters of questionable value, it is difficult for wider applicability in routine clinical practice.

**Table 2:** Revised original diagnostic scoring system of the International Autoimmune Hepatitis Group in 1999

No.	Clinical feature		Score
1	Female		+ 2
2	ALP/AST (or ALT) ratio		
		< 1.5	+ 2
		1.5–3.0	0
		> 3.0	− 2
3	Serum globulin or IgG level above ULN		
		> 2.0	+ 3
		1.5–2.0	+ 2
		1.0–1.5	+ 1
		< 1.0	0
4		ANA, SMA, or anti-LKM1	
		> 1:80	+ 3
		1:80	+ 2
		1:40	+ 1
		< 1:40	0
		AMA positive	− 4
5		Hepatitis markers	
		Positive	− 3
		Negative	+ 3
6	Hepatotoxic drug exposure		
		Positive	− 4
		Negative	+ 1
7	Average alcohol intake (g/day)		
		< 25	+ 2
		> 60	− 2
8	Histologic findings		
		Interface hepatitis	+ 3
		Lymphoplasmacytic infiltrate	+ 1
		Rosette formation	+ 1
		None of the above	− 5
		Biliary changes	− 3
		Other atypical changes	− 3
9	Concurrent other immune disease		+ 2
10	Other autoantibodies		+ 2
11	HLA DRB1*03 or DRB1*04		+ 1
12	Response to corticosteroids		
		Complete	+ 2
		Relapse after drug withdrawal	+ 3
Aggregate score pretreatment			
Definite AIH			> 15
Probable AIH			10–15
Aggregate score posttreatment			
Definite AIH			> 17
Probable AIH			12–17

*ALP* alkaline phosphatase, *AST* aspartate aminotransferase, *ALT* alanine aminotransferase, *IgG* immunoglobulin G, *ULN* upper limit of the normal range, *HLA* human leukocyte antigen, *ANA* antinuclear antibodies, *SMA* smooth muscle antibodies, *anti-LKM1* antibodies to liver kidney microsome type 1, *AMA* antimitochondrial antibodies

#### Simplified criteria for the diagnosis of AIH [174]

To simplify the use of revised original diagnostic scoring system, the IAIHG defined simplified diagnostic criteria for routine clinical practice in 2008. The simplified score system is a reliable and simple tool to establish and exclude the diagnosis of AIH more frequently in liver diseases concurrent with immune manifestations, it was purely meant for clinical purposes [174] (Table 3). The simplified score system has superior specificity and accuracy comparing to the original revised scoring system [174], but only includes four clinical components, and no treatment response in the scoring system, it is generally accepted that simplified score system has a lower sensitivity [175]. However, it is exciting to find that in Asia, the simplified criteria for the diagnosis of AIH has higher sensitivity and specificity than the revised original diagnostic scoring system. In Chinese AIH patients, the sensitivity and specificity were 90 and 95% for the probable AIH, and 62 and 99% for definite AIH [176]. In Japan, the sensitivity and specificity of simplified diagnostic criteria for AIH was 85 and 99%, respectively, in diagnosis of the AIH [173]. In Korea, the diagnostic sensitivity and positive predictive value of the simplified diagnostic criteria were 69.9 and 86.4%, respectively [177]. The simplified criteria are generally useful for the diagnosis of AIH with typical AIH features, but it will be not applicable for patients with atypical features, such as serum IgG levels under the upper normal limit, ANA or SMA titres less than 1:40, and patients with acute hepatitis who need to start immunosuppressive treatment [173]. When the score of the simplified scoring system is lower than 6, the revised scoring system should be considered to further determine whether there is AIH.

**Table 3:** Simplified criteria for the diagnosis of AIH

	Clinical feature	Result	Score
1	ANA or SMA	≥ 1:40 by IIF	+ 1
	ANA or SMA	≥ 1:80 by IIF	+ 2*
	Anti-LKM1 (alternative to ANA and SMA)	≥ 1:40 by IIF	+ 2*
	Anti-SLA (alternative to ANA, SMA and anti-LKM1)	Positive	+ 2*
2	IgG	> UNL	+ 1
		> 1.1 UNL	+ 2
3	Liver histology	Compatible with (evidence of hepatitis is a necessary condition)	
		AIH	+ 1
		Typical AIH	+ 2
		Atypical AIH	0
4	Absence of viral hepatitis	Yes	+ 2
		No	0
		Total scores	≥ 6: probable AIH
			≥ 7: definite AIH

*Sum of points achieved for all autoantibodies (maximum 2 points)

### Diagnosis of AIH-PBC overlap syndrome

“Paris criteria” is the most common and effective method used to diagnosis the AIH-PBC overlap syndrome. It requires at least two of the following three diagnostic criteria for each disease: for diagnosis of PBC: (1) Serum alkaline phosphatase (ALP) levels at least two times the upper limit of normal (ULN) or serum gamma glutamyl transferase (GGT) levels at least five times ULN; (2) Presence of anti-mitochondrial antibodies (AMA); (3) A liver biopsy specimen showing florid bile duct lesions. For AIH, it requires two of the following three diagnostic criteria: (1) Alanine aminotransferase (ALT) activity > 5 times ULN; (2) IgG ≥  2.0 times ULN and/or positive SMA; (3) Liver biopsy with moderate or severe interface hepatitis [178].

“Paris criteria” is a classic method for diagnosing AIH-PBC overlap syndrome. In China, the specificity of Paris criteria for AIH-PBC overlap syndrome was 100%, but the sensitivity was only 10%. Ma’s research group reported, in Chinese AIH patients when modified the Paris criteria by using a lower threshold, serum IgG levels as ≥ 1.3 ULN, the diagnostic sensitivity and specificity had been improved to 60 and 97%, respectively [179]. Except for Paris criteria, the revised original Diagnostic scoring system of the IAIHG and simplified AIH scoring system have also been applied to determine overlap syndrome, but the former were designed for the diagnosis of AIH with the exclusion of PBC, it was not an optimal diagnostic criteria, as for simplified AIH scoring system, it has been gradually accepted for diagnosing PBC–AIH overlap syndrome, especially in Chinese patients [166].

### Diagnosis of AIH-PSC or IgG4-related SC overlap syndrome

AIH-PSC overlap syndrome is regarded as a variant of PSC. It is uncommon, but when PSC patients present some clinical features of AIH, AIH-PSC overlap syndrome need to be excluded. The diagnosis of PSC is based on the alteration of cholestatic enzymes, the typical changes of the biliary tree with multifocal strictures and segmental dilatation by magnetic resonance cholangiopancreatography (MRCP) or direct cholangiography (ERCP) [180]. In 2000, Mayo clinic reported the revised AIH scoring system seems to be more precisely for the diagnosis of AIH-PSC [181]. In 2017, Ma’s group reported their data on 148 PSC patients, in these patients, when used the simplified criteria of AIH, 36 AIH-PSC overlap syndrome diagnosis was established, when serum IgG4 level was more than 1.25 ULN, it would be helpful to differential diagnosis IgG4-SC among SC patients [182].

### Differential diagnosis of AIH

AIH usually presents with features of acute or chronic hepatitis, and lacks a specific diagnostic method, sometimes it is difficult to differentiate from other causes of liver diseases, such as DILI, nonalcoholic fatty liver disease (NAFLD), viral hepatitis, or hereditary metabolic liver diseases et al.

#### DILI

DILI, especially the idiosyncratic DILI has features similar to those of other liver diseases including AIH. Idiosyncratic DILI can mimic the clinical features of AIH, the circulating autoantibodies and a hypergammaglobulinemia are frequently present in sera, even the hepatic histology demonstrate interface hepatitis with a prominent plasma cell infiltrate. Drug may serve as a trigger for induction of persistent AIH. Distinguishing DILI from AIH can be extremely difficult in these patients, a liver biopsy should be recommended to determine the diagnosis [113]. A response to corticosteroid therapy and a lack of recurrence of symptoms or signs following corticosteroid cessation can distinguish AIH-DILI from idiopathic AIH [183], and support DILI diagnosis.

#### NAFLD or concurrence with steatohepatitis

NAFLD has some similar clinical and histological features with AIH. Low titers of autoantibodies (ANA, SMA and/or AMA) and increased γ-globulins, can appear in part of NAFLD patients [184]. In the histological features, lobular inflammation and hepatocyte ballooning can present both in AIH and NAFLD, by using the simplified scoring system for AIH, these features may be misdiagnosed NAFLD as having probable AIH [150] [174]. Furthermore, in the corticosteroid-treated AIH patients, corticosteroid-related side effects, insulin resistance or dyslipidemia can induce the development of fatty liver [66]. Though differential diagnosis of AIH and NAFLD is difficult, the levels of hepatobiliary enzymes (ALT, AST, ALP, GGT) in AIH patients with NAFLD were lower, and the lobular inflammation was less compared to the pure AIH patients [185].

#### Viral hepatitis or concurrence with viral hepatitis

Acute onset of AIH can present acute viral hepatitis like illness especially in absence of autoantibodies and hypergammaglobulinemia. It is necessary to complete serological testing to exclude hepatophilic virus infection before the diagnosis of definite or probable AIH.

For untreated AIH, acute hepatitis virus A (HAV) and acute hepatitis virus E (HEV) infection are common. Acute HAV infections were higher in treatment-naive pediatric AIH patients. [186], and acute HEV infections were higher in treatment-naive adult AIH patients [187]. For the AIH patient undergoing the conventional immunosuppressive therapy, who has persistent abnormal liver function test results, chronic hepatitis E needs to be excluded, and testing for HEV RNA is recommended [187].

ANA, SMA and anti-LKM-1 are commonly detected in the sera of patients with chronic hepatitis B (CHB) or chronic hepatitis C(CHC) [188] [189]. Only 0.83% of Chinese AIH patients were infected by HBV. It seems, HBV in Chinese AIH patients is easier to be cleared [190]. In India, ANA and SMA positivity rate in CHB patients were 27.1 and 25.7%, respectively, while in CHC patients, the positivity rate was 26.9, 46.1 and 11.1% for ANA, SMA, and LKM, respectively. HBV infection in Indian may induce AIH type I, and chronic HCV infection usually causes AIH-Type 2 [191]. The diagnosis of AIH is challenging in these patients because of low sensitivity and specify of AIH scoring systems. In these patients, a definitive diagnosis of AIH should be based on a combination of serological profiles, histological findings, scoring systems, treatment response, and outcomes [99, 192]. The resolved HBV can be reactivated during immunosuppressive therapy for AIH, treating HBV before immunosuppressive therapy or closed follow-up during immunosuppressive therapy is vital.

#### Concurrence with other chronic liver disease

Hereditary metabolic liver disease, such as Wilson disease [193], hemochromatosis [194] can present the features of AIH, and in some AIH patients, laboratory tests showed elevated serum ferritin level, even have a heterozygous C282Y mutation [195].

#### Concurrence with other autoimmune liver diseases

AIH patients may show the cholestatic biochemical profile, and even present some serological markers of PBC, PSC and IgG4-related cholangitis [182, 196, 197], these made the diagnosis of AIH more difficult. Liver biopsy and biliary tree imaging should be recommended in these AIH patients to highlight the predominant liver disease and choose the appropriate treatment regimens [120].

### Guidance

1. The diagnosis of AIH should be based on the autoantibodies (standard autoantibodies-ANA, SMA and Anti-LKM1), elevated IgG or serum globulin, interface hepatitis, and exclusion of other causes of liver disease.
2. Circulating autoantibodies can be negative in part of AIH patients, especially whom in acute AIH or during corticosteroid treatment.
3. Transient elastography can be routinely used for AIH patient either in diagnosis at onset or during the immunosuppressive therapy follow-up.
4. In Asia–Pacific region, the simplified criteria for the diagnosis of AIH has higher sensitivity and specificity, it can be used to diagnosis AIH, AIH-PBC and AIH-PSC/IgG4-related SC overlap syndromes.

## Treatment

### Aim

The aim of AIH treatment is to achieve complete biochemical and histological resolution, to suppress inflammatory activity, to prevent fibrosis progression and onset of end-stage events, eventually to prolong survival and improve life quality of patients.

AIH is a chronic and progressive hepatitis, and could promptly progress to cirrhosis if untreated. Several randomized controlled studies were conducted in the 1960s and 1970s [198–202], and a systematic review of these randomized trials was published in 2010 [203]. Among them, Cook et al. conducted a randomized, controlled, prospected clinical trial in patients with active chronic hepatitis between prednisolone monotherapy (15 mg/day) and placebo. After 72 months of observation, three out of 22 patients treated with prednisolone (14%) and 15 out of 27 patients with placebo (56%) had died [198]. The mean value of AST, serum bilirubin and albumin in placebo group was 118 IU/L, 3.8 mg/dL and 3.0 g/dL, respectively, indicating that AIH patients with decompensated cirrhosis have a high mortality, 56% at 6 months, while intervention with prednisolone decreased mortality to 14%. Another placebo-controlled trial was performed by Soloway et al., in which 17 patients with chronic active hepatitis served as a placebo control [200], and again high mortality (41% died up to 3.5 years) was noted. Even without cirrhosis at presentation, AIH patients with bridging necrosis or multilobular necrosis are likely to progress to cirrhosis in a few years if untreated, and long-term prognosis is poor [204, 205].

On the other hand, the natural history of asymptomatic AIH patients with mild laboratory and histological abnormalities remains largely unknown [66]. Feld et al. demonstrated that AIH patients with asymptomatic at presentation, who had lower serum aminotransferase, bilirubin and IgG, had a good prognosis with 80.0% of 10-year survival even though half of these patients received no treatment, while patients with cirrhosis at baseline exhibited poorer 10-year survival (61.9%) [52]. Nevertheless, 80% of the 10-year survival does not appear to be excellent currently since 10-year survival of patients with AIH exceeds 90% [206]. Furthermore, there have been no biomarkers or histological findings to identify “safe” AIH patients who do not require corticosteroids therapy. It should be noted that the mild AIH can progress to severe fibrosis, leading to poor outcomes.

Recent study, however, suggested the long-term outcome of AIH is now excellent if patients are appropriately managed. In a retrospective cohort study in Japan consisting of 203 patients who were treated with immunosuppressive agents, the overall survival was comparable to those of the general population and 10-year survival was more than 90% [206]. Of note, fibrosis staging at baseline and onset type (acute or chronic) was not associated with the prognosis. Therefore, it is important to bring about biochemical resolution with immunosuppressive treatment for achievement of the improved long-term outcome in AIH.

Biochemical resolution is defined as reduction of transaminase and IgG levels to normal. A clinical study from India [207] showed early treatment coincides with favorable long-term prognosis and failure to normalize alanine aminotransferase is a risk factor predicting disease related mortality or transplantation. A retrospective study demonstrated that more than 50% patients who achieved transaminase level below twice ULN still underwent histological progress [208]. On the other hand, it has been justified that escalating IgG levels are also associated with liver inflammatory activity in AIH patients [209]. Therefore, normalization of both transaminase levels and IgG levels have been considered as the markers of complete biochemical remission [66]. A recent clinical investigation in 120 AIH patients suggested that complete biochemical remission is not only a conceivable surrogate marker for histological disease activity as evidenced by liver biopsies, but also a predictor for the promising prognosis, because the resolution of inflammation leads to fibrosis regression [79]. Moreover, patients with a rapid biochemical response at 8 weeks post-therapy also have a lower risk of liver-related death or transplantation [210]. To assist physicians to establish therapy schemes, we advocate to determine efficacy of medications by examining serum alanine transaminase and IgG levels at 6 months after treatment [211].

Besides, psychological comorbidity of patient is another important issue that needs early recognition and prompt intervention. It was reported that a major depressive syndrome and severe symptoms of anxiety were found to be significantly more frequent in AIH patients compared to the general population [212], which contributed to an increased risk of noncompliance to AIH therapy [213].

### Indications of treatment

In 2010, the AASLD recommended either of following criteria is absolute indication for corticosteroid treatment [66]: serum ALT/AST level above tenfold ULN, more than fivefold ULN plus serum IgG level more than twice ULN, histological features of bridging necrosis or multilobular necrosis, incapacitating symptoms. In addition, asymptomatic patients with mild laboratory and histological disorder may be considered for treatment. In 2011, the British Society of Gastroenterology (BSG) guidelines proposed treatment in all cases with the serum aminotransferases more than fivefold ULN or serum globulins level at least two times ULN or liver biopsy showing confluent necrosis [39]. In 2015, however, EASL expanded indications to all patients with active AIH [12]. These three guidelines issued at various time reflected the tendency that the treatment threshold was decreasing.

AIH is characterized as a fluctuating and unpredictable inflammation in liver, which leads to end-stage liver diseases, cirrhosis or hepatocellular carcinoma [52, 53, 214]. Therefore, suppression of hepatic inflammation is pivotal to prevent disease progression and improve prognosis [215–217]. Given serum aminotransferases as faithful surrogate markers for hepatic inflammation, it is recommended that all patients with elevated serum aminotransferases should receive treatment [218]. But still a fraction of patients display inflammatory activity on liver histology despite normal aminotransferases [79, 209]. Among them, elevated IgG levels can also reflect ongoing inflammatory activity [209]. Besides, transient elastography is regarded as an applicable non-invasive tool to assess liver inflammatory edema and fibrosis with a high accuracy and repeatability [97, 98]. Finally, liver biopsy is an efficacious strategy to evaluate histological inflammation and fibrosis albeit concomitant risks of sampling error and severe complications [219]. If patients in spontaneous remission cannot accept liver biopsy, the monitoring must be closely followed up every 3–6 months [12]. In sum, patients whose manifestations fulfill either of following criteria—increased IgG levels, enhanced liver stiffness, abnormal histological activity—should be considered for treatment.

### Standard treatment

#### Standard treatment modalities

The 5-year survival rate of 82% in patients treated with steroids compared to 32% in untreated patients highlighted the efficacy of steroids therapy, because it was proved to suppress inflammation and improve liver biochemical abnormalities [220]. Another clinical investigation demonstrated 57% (13/23) patients avoided liver transplantation as a result of the response to corticosteroid-based therapy [221]. The current immunosuppressive treatment modality mostly originates from the studies published in the 1970s [198–200]. An influential comparison study was performed between prednisone monotherapy, azathioprine monotherapy, combination therapy and placebo. There was a mortality benefit from prednisone monotherapy or combination therapy when compared to placebo (6 vs 7 vs 41%). These two regimes had also similar beneficial effects from histological, biochemical and clinical profiles, but the combination regime was associated with fewer side effects than prednisolone monotherapy (10 vs 44%). On the other hand, azathioprine monotherapy resulted in a higher mortality (36%) and more adverse events (30%) compared to prednisone monotherapy [200]. Thus, the combination therapy of prednisone and azathioprine acted as the frontline regimen.

In 2010, AASLD guidelines [66] suggested starting treatment with prednisone alone (60 mg daily) or with a lower dose of prednisone (30 mg daily) plus azathioprine (50 mg daily), then tapering down over 4 weeks to 20 mg daily which is further continued to reduce until treatment endpoint. Based on BSG guidelines in 2011 [39], 30 mg daily of prednisone is introduced and 5–10 mg daily is maintained in the end. EASL clinical practice guidelines recommended the proposed dose of prednisone in initiating at 60 mg/day and reducing to 20 mg over 6 weeks combined with azathioprine of 1–2 mg/kg [12]. Since the rapid biochemical response is predictive of a low frequency of fibrosis progression and a decreased mortality [222], a retrospective study from Turkey showed starting prednisolone dose of 40 mg daily and tapering over 9 weeks in partnership with azathioprine were more favorable compared to the initial 30 mg daily with a faster dose reduction protocol in 3-month biochemical response (69.2 vs. 43.8%, *p* = 0.031) and 12-month overall survival (100 vs. 87.5%, *p* = 0.048), but not 6- and 12-month biochemical response (79.5 vs. 59.4%, *p* = 0.065 and 89.5 vs. 80.6%, *p* = 0.30), and relapse ratio (35.9 vs. 50%, *p* = 0.23). Meanwhile, no severe prednisolone-related side effects were identified in either group [223] (Table 4). A nationwide survey in 1292 Japanese patients revealed the initial and maintenance dosage of prednisolone that most patients took were 30–40 mg daily and 5–7.5 mg daily, accounting for 39 and 50.6%, respectively [149]. The evidence from China suggested that HLA-DR4 is the predominant disease-susceptible gene and clinical manifestations of those patients are milder, a lower initial dose with 0.5–1 mg/kg daily of prednisone can achieve a satisfactory response [1]. A meta-analysis of 25 studies containing 3305 patients, demonstrated that 60 mg/day or 1 mg/kg/day of glucocorticoid achieved higher levels of biochemical remission, yet also caused more side effects incidence compared with the low dose (40–50 mg/day or 0.5 mg/kg/day) group (79 vs. 72% and 42 vs. 39%, respectively) [224]. A recent European multi-center study showed that overall remission induction rates after 6 months of therapy were similar in patients treated with either high- or low-dose (≥ 0.50 vs. < 0.50 mg/kg/day, respectively) prednisone and advocated an initial low dose prednisone for the treatment of AIH [225]. And the starting dosage could be further lowered to 20 mg daily if incorporation with azathioprine 50–150 mg daily. Indeed, we prefer 50 mg daily for azathioprine to prevent its adverse effects in Asian population empirically. On the other hand, we also encourage to start it until 2 weeks after usage of prednisone to confirm steroid responsiveness, because the discontinuation rate of azathioprine could reach 15% in the first year of treatment [226]. Taken all together, we proposed the treatment regimen is dependent on the severity of disease. The treatment protocol is shown in Table 4 if fulfilling either of the following criteria: serum ALT/AST level above tenfold ULN, more than fivefold ULN plus serum IgG level more than twice ULN, histological features of bridging necrosis or multilobular necrosis, incapacitating symptoms. Otherwise, relatively low-dosage regimens modality is a better treatment option for the patients with mild disease behavior (Table 4).

**Table 4:** Treatment regimen for AIH patients

Combination therapy		Monotherapy
Prednisone	Azathioprine	Prednisone
20 mg daily × 2 weeks	50–150 mg daily	30–40 mg daily × 2 weeks
15 mg daily × 2 weeks		25–30 mg daily × 2 weeks
10 mg daily × 4 weeks		20–25 mg daily × 4 weeks
5 mg daily maintenance		Tapering till to 5 mg daily maintenance

#### Side effects

Almost half of AIH patients present cosmetic changes or obesity after the usage of steroids [227]. Severe, but less frequent steroid side effects include osteoporosis, diabetes mellitus, cataract, psychosis and hypertension [66]. It was reported that diabetes mellitus occurs in 15–20% of treated AIH patients and the morbidity of hypertension, psychosis, cataract, vertebral collapse related to osteoporosis is between 5 and 10% [199, 200]. To prevent or treat osteoporosis, periodic dual energy X-ray absorptiometry (DEXA) scanning and medical therapy, including supplemental calcium, vitamin D and bisphosphonates, are essential for patients who receive long-term steroid therapy [228]. Side effects occur mostly at dose more than 20 mg daily for more than 18 months and resulted in therapy termination in 15% patients. Therefore, supplementation with azathioprine is warranted to reduce onset of steroids specific adverse events [203]. On the other hand, a recent study demonstrated corticosteroid use is strongly associated with impaired health-related quality of life in AIH patients independently of steroid type, dose and biochemical remission status. This highlights novel corticosteroid-free therapy approaches are required to circumvent corticosteroid-related side effects in AIH [229]. On top of this, a retrospectively collected data on 476 patients suggested a low-dose prednisone (0.1–5.0 mg/day) increased the odds of fractures whereas higher doses (> 5.0 mg/day) increased the odds of cataracts and diabetes. Thus, even low doses of corticosteroids frequently lead to substantial adverse events arguing against the assumption that adverse events are prevented by administering low doses [230].

The most common side effect of azathioprine is bone marrow suppression. Thus, it should be avoided in patients with severe pretreatment cytopenia (white blood cell counts below 2.5 × 10^9^/L or platelet counts below 50 × 10^9^/L) and testing for the genetic polymorphism of two azathioprine-based catabolic enzymes, thiopurine methyltransferase (TPMT) and NUDT15, should be encouraged before treatment. In the Caucasian population, approximately 11 and 0.3% presented heterozygous and homozygous TPMT variant, respectively [231]. In contrast, an investigation on prevalence of TPMT polymorphism from 126 Indian patients found only 4.77% patients carrying heterozygous alteration and none bearing homozygous mutation [232], as with Chinese and Japanese patients [233, 234]. In search of other factors contributing to the higher incidence of adverse reactions in Asian patients [235], p.Arg139Cys mutation in NUDT15 was firstly reported to associate with azathioprine-induced early leukopenia in Koreans [236]. Then, recent evidences suggested that it was detectable in 4.4–10.5% of the populations from Asian countries [237–239]. For homozygous TPMT or NUDT15 variation with low concentration of enzyme, azathioprine should be contraindicated, because it will lead to severe cytopenic and septic complications. For heterozygous mutation, it should begin at a lower dose with monitoring of white cell count during treatment [66, 240, 241]. Another possible complication of long-term treatment with azathioprine is the development of malignancies at a dose of 2 mg/kg/day [242]. Moreover, azathioprine has its own profile of toxic effects, such as nausea, vomiting, rashes, pancreatitis and hepatotoxicity [243, 244]. Thus, EASL recommended that it should be introduced after 2 weeks of prednisone alone to distinguish between azathioprine-induced hepatotoxicity and nonresponse to prednisone particularly in cirrhotic and jaundiced patients [245]. Of note, its side effects more commonly take place in cirrhotic patients. About 5% of patients develop one manifestation such as arthralgias, fever, rash or abdominal pain which might require prompt discontinuation [246]. Long-term azathioprine therapy in a large number of pregnant female were found no increase in the risk of low birth weight or teratogenicity [247]. Therefore, azathioprine was approved to continue throughout pregnancy by 2019 AASLD guidelines [248]. Moreover, it is regarded safe for breastfeeding although small amount of metabolite can be quantified in breastmilk [249].

#### Duration of treatment

Most of AIH patients respond well to frontline regimen and achieve biochemical normalization [222, 250, 251]. But relapse after treatment withdrawal becomes a major issue. One study of 30 patients who had been in remission for between 1.5 and 9 years suggested only three remained improvement in 1 year after treatment withdrawal, and ultimately all relapsed [252]. Other studies showed that 50–90% of patients relapsed after 3-year post-discontinuation [253, 254]. A clinical study from Turkey also pointed out relapse is very frequent and only 4.2% patients were off immunosuppressive treatment finally [223]. Another study suggested that normal liver biochemistry (ALT levels less than half the ULN and IgG levels not higher than 12 g/L at the time of treatment withdrawal) can predict success rates of permanent immunosuppressive withdrawal. However, these results need to be validated further [255]. Because most of the reports underlined relapse is quite common after treatment discontinuation and current immunosuppressive therapy only represses inflammation temporarily and the histological improvement lags greatly behind lab test improvement, most patients need the long-term treatment except cirrhosis and significant fibrosis because they will bring forth more severe side effects [256]. As encountering a public health emergency, the telehealth system is as effective and useful in the management of AIH as in regular time [257].

Regarding the previous criteria about treatment endpoint, AASLD guidelines [66] mentioned the termination of treatment might be considered after at least 2-year course only for the patients with biochemical and histological normalization. BSG guidelines [39] proposed re-biopsy is advisable in 1–2 years after serum transaminases normalization. Even histological remission presents, maintenance strategy still needs to be implemented. EASL guidelines [12] recommended that treatment should proceed for at least three years and for at least 2 years after complete normalization of serum transaminases and IgG levels. For patients with severe manifestation and low tolerance to treatment, a liver biopsy before treatment withdrawal should be performed. These guidelines collectively suggested the criteria of treatment endpoint should be stringent. Therefore, we propose the treatment withdrawal can be taken into consideration only in the context of complete biochemical remission for at least 2 years [12] or histological examination shows a alleviated histological disease activity (HAI > 3), is achieved [12, 209], because histological examination is more accurate to evaluate the real status quo in liver and predict fibrosis progression and relapse.

During the course of treatment, efficacy parameters need to be monitored as shown in Table 5. If either serum aminotransferases or IgG titers cannot improve, the prednisone dose should maintain in conditions of below 20 mg daily as a stand-alone therapy. Notably, transient elastography can be used to measure the liver stiffness reflective of the severity of inflammatory edema or fibrosis [12]. Furthermore, the likelihood of HCC development calls for periodic workup of abdominal ultrasonography and tumor markers [70].

**Table 5:** Treatment efficacy monitoring program

Variable	Induction therapy	Maintenance therapy
Liver biochemistry	Every 1–3 months	Every 6 months–1 year
Serum IgG	Every 1–3 months	Every 6 months–1 year
Serum autoantibodies	Every 3 months	Every 6 months–1 year
HCC-associated tumor markers	Every 6 months	
Abdominal ultrasonography	Every 1 year	
Transient elastography	Every 1 year	
Liver biopsy	Non-response, incomplete response, before the treatment withdrawal	

#### Management to difficult-to-treat patients

A lack of a reduction of transaminases by more than 25% in 2 weeks or a worsening of coagulation markers or bilirubin levels should be characterized as non-response [12]. In this case, three possibilities need to be excluded, i.e., non-compliance, analogous diseases with AIH, such as viral hepatitis, Wilson’s disease, NASH, DILI, PSC or PBC, and the concomitant cholestatic syndrome that may be refractory to the original treatment. Liver biopsy might favor to distinguish following reconfirmation of adherence. For AIH patients with non-response, dosage of prednisolone and azathioprine should be increased or alternative medications should be implemented [258]. One strategy is to increase the dosage of prednisone to 60 mg daily or increase the azathioprine dosage to up to 150 mg daily in combination with prednisone 30 mg daily for 2 weeks [66]. In concurrent regimen, 6-TGN, the metabolite of azathioprine, should be measured regularly to evaluate patient’s compliance and avoid toxicity [259].

For AIH patients with incomplete response, if any effort to normalize transaminases is not achievable, it should be adjusted to maintain transaminase level below threefold greater than upper limit of normal to reduce the likelihood of aggressive interface hepatitis and progression of the disease [260].

It is estimated that 10–15% of patients on standard therapy discontinued treatment due to intolerable side effects [261]. For patients with drug intolerance, alternative treatment strategies can be applied. For patients with intolerance of steroid induced side effects, a shift to budesonide with 6 mg daily or higher doses of azathioprine (2 mg/kg) may be applicable; or an alternation to Mycophenolate Mofetil (MMF) with 2 g daily and subsequent tapering of steroids is also feasible in conditions of restriction of azathioprine dose due to drug toxicity or side effects [262]. For patients intolerant to azathioprine, MMF with 2 g daily tends to be preferable. 6-MP is another consideration for patients with obvious azathioprine intolerance or other second-line nonsteroidal regimens can also be tried in this case [246].

**Fig. 2:**
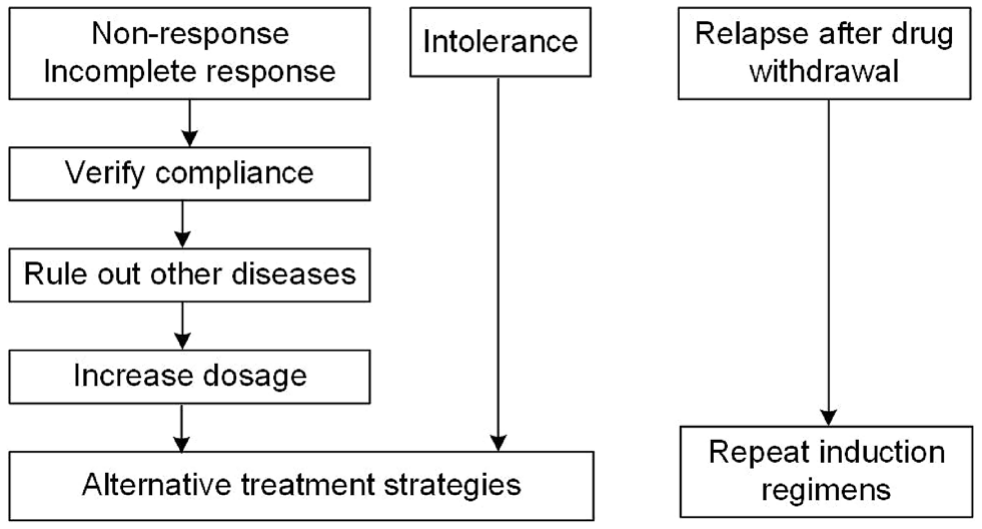
Treatment strategy to difficult-to-treat patients with AIH

Disease relapse is defined as transaminase levels rising abnormally after remission [263]. As mentioned above, it is very common after treatment withdrawal in AIH patients. In this regard, prednisone and azathioprine doses are required similar to primary induction regimen [264]. The summarized treatment strategy to difficult-to-treat patients is shown in Fig. 2.

#### Management to special types of AIH

##### Acute presentation

Combination therapy with prednisone and azathioprine can result in clinical and laboratory improvement in 68–75% of patients with acute presentations, but the use of prednisone in acute severe-AIH, which is defined as an acute presentation of the illness (less than half year between symptom onset and presentation) and an INR ≥ 1.5 in absence of cirrhosis, remains controversial, because there are not identified and evident benefits [126, 265]. Given the urgency of inflammation and limitation of donor livers, patients should be considered for a trial of prednisolone (≥ 1 mg/kg) at the earliest opportunity [12, 64], although higher levels of bilirubin (> 10 mg/dL) and a MELD score (> 28.5), as well as an elevated INR value (> 2.46) are poor response markers to prednisolone therapy [137, 266]. A retrospective study in 22 Chinese AIH cases found younger age and earlier glucocorticoids administration are beneficial factors for survival [267]. In the midst of treatment, the risk of infections needs the administration of prophylactic antibiotics and antifungal agents, although it remains questionable whether the sepsis impairs the outcome of treated patients [268]. Those who lack improvement of serum bilirubin and MELD score after 2 weeks of prednisolone treatment should consider other therapeutic methods, particularly liver transplantation [151, 269–271]. The initial median dose of 60 vs. 40 mg/day induced comparable response from two clinical trials. This suggests that moderate corticosteroid dosing may be sufficient for treatment. Moreover, a similar number of treated patients developed sepsis in both studies (20 vs. 26%) [221, 266]. Thus, we recommend prednisolone (40 mg daily) could be applied for 2 weeks initially in acute severe-AIH context.

The utility of steroid in flare of chronic AIH remains to be a further investigation. A recent study from the Asian Pacific Association for the Study of the (APASL) Research Consortium (AARC) indicates that AIH patients with acute-on-chronic liver failure (ACLF) received treatment with prednisolone (40 mg daily) for 1 month followed by a tapering dose, leading to shorter ICU stay (1.5 vs. 4 days, *p* < 0.0001) and improved 90-day survival (75 vs. 48.1%, *p* = 0.02), yet indistinguishable incidence of sepsis in contrast with untreated patients. Patients with advanced age, severe liver disease (MELD > 27), hepatic encephalopathy or fibrosis grade above F3, however, exhibited unfavorable effects to prednisolone therapy [62]. Patients with liver failure should consider liver transplantation.

##### Decompensated cirrhosis

Prednisolone is appropriate for AIH patients at the decompensated cirrhosis stage. In the corticosteroid-treated group, 62.5% (40/64) patients were reversed to the compensated state [151]. Two Chinese clinical investigations [272, 273] demonstrated the efficacy of initial immunosuppressive treatment in AIH patients with cirrhosis is comparable to that in those without cirrhosis. Cirrhotic patients untreated by immunosuppressive therapy have poor long-term outcomes. Due to impaired liver metabolic function at cirrhosis, prednisolone is preferable for the advanced cirrhosis stage. However, the benefits should be counterbalanced with the high risk of gastrointestinal bleeding and infection, and adjunctive therapies, such as antacids and sympatholytic nonselective beta-blockers should be applied [151, 274]. On the other hand, there are a few studies [275, 276] which have shown liver fibrosis is an independent predictor of non-response to corticosteroids. HCC incidence is another concern for cirrhotic patients. According to statistics, the morbidity for HCC in patients with AIH was 3.06 per 1000 patients, whereas the incidence of HCC in cirrhotic patients at AIH diagnosis was 10.07 per 1000 patients. However, it is still less common than that reported for patients with cirrhosis from hepatitis B, hepatitis C, or primary biliary cholangitis [69, 277]. Thus, ultrasound and tumor markers examinations should be conducted more frequently [70].

#### Management to special populations

##### Pregnancy

The maternal complication rate during the pregnancy of mother with AIH is 38%, wherein prematurity is mainly due to withholding from adequate treatment [278]. Therefore, pregnant patients with AIH need to receive continuous treatment to reduce the risk of flare and hepatic decompensation [279]. The use of prednisone is regarded as safe for pregnant female and fetal after a case–control study revealed no association with neonatal cleft lip by US National Birth Defects Prevention [280]. Meanwhile, azathioprine therapy in fertile young adults did not amplify the risk of preterm birth and teratogenicity [248]. Thus, sustained prednisone and/or azathioprine therapy is necessary to reduce the odds of flare and maternal complications. Noticeably, flares are three times more prevalent after delivery [281], which underscore the need for closer monitoring and follow-up of patients.

##### Children

In the time of diagnosis, more than 50% of children will have evidence of cirrhosis, and the milder forms of disease are scanty. This requires initiation of early treatment following diagnosis [282, 283]. In children, the recommended treatment protocol is similar to that of adults, but a higher steroid dose is warranted due to the more grievous disease course [282, 283]. Starting dose of prednisone at 1–2 mg/kg daily and early administration of azathioprine (1–2 mg/kg daily) is preferred unless contraindications exist [284]. Meanwhile, the treatment duration is more likely to maintain in the long term because the relapse rate is 46% in adults and 80% in children patients after drug withdrawal in satisfaction of the remission criterion for more than 2 years. Furthermore, longstanding biochemical remission is possible in 20% of children with type 1 AIH, but scarce in children with type 2 AIH [100]. In a Turkish study of 47 children, corticosteroid was started at 2 mg/kg daily, then was reduced gradually at 3.6 ± 2.8 months. Maintenance therapy with oral low-dose corticosteroid (5 mg daily) and azathioprine (2–2.5 mg/kg daily) determined that 37 patients (88%) achieved a CR, and 3 patients (9.4%) relapsed at 8, 12, and 48 months [242].

##### Elderly

Elderly patients are more likely to maintain remission than younger patients after treatment, but treatment in the elderly should be based on the strict criteria due to drug-related side effects, particularly under high dose of prednisone [285]. Benefits from treatment of old patients with mild disease activity are negligible, because 10-year survival has been reported to range from 67 to 90% even without treatment [214]. However, AIH manifests as a fluctuating inflammation which is bound to result in cirrhosis and hepatocellular carcinoma. Therefore, management of patients with mild disease activity is optional with a dependency on comprehensive balance between all risks and benefits. For those untreated patients, monitoring should be closely carried out to measure transaminases and serum IgG every 3 months [12]. Elderly patients with liver failure or hepatocellular carcinoma should seek for liver transplantation if they have good functional status and no significant comorbidities [286].

### Guidance on treatment

1. The aim of AIH treatment is to achieve complete biochemical and histological resolution to prevent further progression of disease.
2. All patients with elevated serum aminotransferases, increased IgG levels, enhanced liver stiffness or abnormal histological activity should be considered for treatment.
3. The initial dose of prednisone should be 30–40 mg daily or 20 mg daily along with 50–150 mg daily of azathioprine.
4. Periodic DEXA scanning and supplementation of Vitamin D and adequate calcium should be recommended to all patients receiving steroid therapy.
5. Incorporation with azathioprine is warranted to reduce onset of steroids specific adverse events.
6. Bone marrow suppression need be noticed in the patients treated with azathioprine, among which the evaluation of TPMT polymorphism is recommended before treatment.
7. Treatment with prednisone and/or azathioprine therapy is necessary to be maintained during pregnancy.
8. Because underlined relapse is quite common after treatment discontinuation, most patients need the long-term treatment. Only patients in spontaneous remission may not require therapy but must be closely followed up.
9. Liver biochemistry, serum IgG, autoantibodies, HCC associated tumor markers, abdominal ultrasonography and transient elastography need to be monitored regularly during the course of treatment.
10. Liver biopsy might favor the differential diagnoses for AIH patients with non-response.
11. For patients with drug intolerance, alternative treatment strategies can be applied, such as budesonide, MMF and 6-MP.
12. Prednisone and azathioprine doses are required similar to primary induction regimen upon the treatment of disease relapse.
13. Acute severe-AIH patients should be considered for prednisolone (40 mg daily) treatment for 2 weeks initially. The failure to respond would be a cut-off point to withdraw this therapy.
14. AIH patients with ACLF received treatment with prednisolone (40 mg daily) for 1 month followed by a tapering dose, leading to shorter ICU stay and improved 90-day survival.
15. The efficacy of initial immunosuppressive treatment in AIH patients with cirrhosis is comparable to that in those without cirrhosis.
16. A higher starting dose of prednisone at 1–2 mg/kg daily and early administration of azathioprine (1–2 mg/kg daily) are preferred in children with AIH due to the more grievous disease course.
17. Elderly patients are more likely to maintain remission than younger patients after treatment, but treatment should be based on the strict criteria because of drug-related side effects.

### The alternative treatment of AIH

Not all patients respond to conventional treatment with prednisone and azathioprine, and those who do respond may develop side effects related to the treatment or relapse after drug withdrawal. Suboptimal responses in patients with AIH include treatment failure, incomplete response, drug toxicity and relapse after treatment withdrawal [218]. While there is consensus on the ideal first-line therapies for AIH, there is little agreement regarding the treatment of patients with suboptimal responses [12]. The AASLD [66] suggests that failure to conventional therapy should be initially managed with high doses of prednisone before considering other therapies. EASL considers, although the alternative treatments are widely used, RCT trials are lacking. All of them were performed by experience [12]. Tacrolimus and prednisone acquired the highest average rate of improvement in aminotransferases level (94.3%), whereas the average improvement rates in cyclosporine and prednisone, budesonide and mycophenolate, prednisone are 91.3, 85.5, and 78.7%, respectively. The respond rate of the aminotransferases ranges between 78.7 and 94.3% [287].

#### Budesonide

Budesonide is the second generation of corticosteroid, has an affinity 15 times than that of prednisone. It can be used as first-line and alternative treatment. Oral budesonide has a relatively high concentration in the hepatic cell before elimination, thus obviously reducing the systemic side effect [288]. A decreased liver function presents as lowered albumin, elevated bilirubin, and lowered prothrombin time and results in a higher systemic budesonide concentration. In the presence of portal hypertension and portocaval shunting, as seen in cirrhosis, the systemic concentration of budesonide is even higher. Cirrhotic patients were excluded, because the first pass hepatic extraction of budesonide may be reduced in cirrhosis due to portosystemic shunting [218]. A retrospective study of Iman Zandieh et al. [288] included nine patients of AIH, the indications for budesonide were adverse side effects of prednisone in two patients, noncompliance with prednisone and azathioprine in one patient and intolerance to azathioprine resulting in prednisone dependence in the remaining six patients. Patients were treated in doses ranging from 9 mg daily to 3 mg every other day for 24 weeks to 8 years. Seven of nine patients had a complete response (CR), defined as sustained normalization of the aminotransferase levels. The side effect involves abdominal pain, weight gain, acne, hair loss and Cushing face, only occurred in cirrhotic patients, because the metabolism of liver decrease [289]. In non-cirrhotic patients, moon-face, acne, hirsute are most frequent seen [290]. In a 6-month, prospective, double-blind, randomized, active-controlled, multicenter, phase IIb trial of patients with AIH without evidence of cirrhosis, patients were given budesonide (3 mg, three times daily or twice daily) or prednisone (40 mg/day, tapered to 10 mg/day), with azathioprine at the same time (1–2 mg/kg/day). The primary endpoint (complete biochemical remission, defined as normal serum levels of AST and ALT, without predefined steroid-specific side effects) was achieved in 47/100 patients given budesonide (47.0%) and in 19/103 patients given prednisone (18.4%). For non-cirrhotic patients, the corticosteroid-induced side effect of budesonide obviously rare than pre [291], so the budesonide is suitable for those treatment-naïve, non-cirrhotic, without complex diseases, who are also in high risk of corticosteroid-induced side effect [291–293]. Although budesonide is an attractive treatment strategy for AIH patients without cirrhosis, caution is advised for persistent, vague symptoms which could reflect adrenal insufficiency. Simultaneous intake of other drugs affecting CYP3A4 should be taken into account and should better be avoided [294]. Study shows that the remission rate of budesonide/AZA is higher than that of prednisone/AZA, budesonide in combination with AZA may be appropriate treatment for patients without findings of advanced liver disease [276]. However, budesonide/AZA as frontline therapy in adults with AIH requires additional large-scale studies with a longer duration of follow-up histology and a focus on dose–response [295]. Budesonide is also useful in maintenance [218], but for those who resist or on-respond to pre, budesonide may not be effective, for they share the same mechanism [218, 296].

#### Mycophenolate mofetil (MMF)

MMF (1–2 g/day) is widely used as second-line AIH treatment, mostly combined with prednisone, both for patients intolerant to azathioprine and for patients with unsatisfactory response to standard azathioprine/prednisone treatment [218]. MMF is strictly contraindicated during pregnancy. MMF was studied the most among all the drugs which treat AIH [287]. There are no direct comparisons between different second-line treatments, even retrospective studies. The choice between these drugs often depends on the expert opinions [287]. The most common side effect is gastroenterological symptom [218], side effect includes leukopenia, nephropyelitis, diarrhea, septicemia, neuropsychological symptom, skin rashes and hair loss [297–299]. MMF can be used in cirrhotic patients, in a study 14 (73.6%) patients were still with biochemical remission, including four out of five patients with cirrhosis, main side effect is skin rashes and hair loss[300], 88% (52/59) of patients responded initially clinically and biochemically (normalization of transaminases and γ-globulins) most of them within 3 months. In total, 59.3% (35/59) of patients had CR with 37% (22/59) of them having achieved CR off prednisolone.

MMF seems safe and effective as first-line therapy in inducing and maintaining remission in treatment-naive patients with AIH, having a significant and rapid steroid sparing effect as attested by the fact that so far, 37% (22/59) of AIH patients achieved CR off prednisolone, Overall, the adverse events which were considered to be related to MMF resulting in dose reduction and/or treatment discontinuation were 4/59 (6.8%) [301]. Retrospective multi-center study in 22 patients with AIH who failed azathioprine and prednisolone due to adverse events (64%), lack of remission (23%) or a combination (13%). Normal aminotransferase levels were obtained (*n* = 3) or maintained (*n* = 7) in 10 patients (45%) after 3–30 weeks. 12 patients (55%) were withdrawn during the first 6 months, due to adverse events. Adverse events were nausea, headache, diarrhea, erythema and subcutaneous vasodilatation, especially those with previous intolerance to thiopurines (64%) [302]. A retrospective study of 105 patients with AIH who received mycophenolate mofetil therapy after an inadequate response or intolerance to standard therapy. Overall 63 patients (60%) achieved biochemical remission following a median 12 weeks treatment with mycophenolate mofetil. The proportion of patients who achieved biochemical remission was similar between patients receiving mycophenolate mofetil for non-response to standard therapy (57%) and patients with intolerance to standard therapy (62%). However, a lower proportion of patients with cirrhosis achieved biochemical remission (47%) than patients without cirrhosis (6%) [303]. Another retrospective study (from 19 centers in Europe, the United States, Canada, and China) from 201 patients with AIH who received second-line therapy (121 received MMF and 80 received tacrolimus), long-term therapy with MMF or tacrolimus generally was well tolerated by patients with AIH. The agents were equally effective in previous complete responders who did not tolerate standard therapy. Tacrolimus led to a CR in a greater proportion of previous non-responder patients compared with MMF. There was no significant difference in the proportion of patients with a CR to MMF (69.4%) vs. tacrolimus (72.5%) [304].

#### Cyclosporine A (CsA)

CsA is a calcineurin inhibitor extensively used in the setting of transplant medicine. Important side effects are renal toxicity and cosmetic changes, particularly in association to high doses [218]. A single center RCT study with 39 treatment-naïve patients [305] showed that at week 12, 64.3% patients treated with CsA had achieved AST and ALT in the normal range, and the final (week 48) mean serum creatinine for patients in this group was not statistically different with their baseline values. CsA usually dosed in 2–3 mg/kg/day. Though the results of these reports appear to be encouraging, the quality and quantity of the data are insufficient to recommend its use [218].

#### Tacrolimus

Tacrolimus is a more potent calcineurin inhibitor than cyclosporine, has less cosmetic side effects, but similar drug class toxicity. In a study, significantly more patients given tacrolimus compared with MMF had a CR (56.5 vs. 34%, respectively) [304]. A meta-analysis with seven articles achieving the inclusion criteria and reported data for a total of 162 adult patients. Treatment duration ranged from 1 to 136 months, and at a dose of tacrolimus 0.5–6 mg/day. Indications for therapy were mostly AIH refractory to steroid treatment or inability to tolerate standard steroid treatment. One hundred and twenty-one patients (74.7%) demonstrated complete biochemical response to treatment. 83.3% histological remission according to the grade of inflammation or stage of fibrosis. Renal function remained stable in most of the patients, thus demonstrating the efficacy of tacrolimus in patients with AIH with minimal side effects. Tacrolimus can be a potential treatment option for patients with AIH refractory to standard therapy [306]. In another study, with 17 refractory patients, the majority of patients achieved biochemical and immunological response with tacrolimus therapy in first year of therapy. None of the patients experienced major side effects or renal dysfunction as a result of Tacrolimus therapy. Serum creatinine level remained stable over 11 years of tacrolimus treatment [307].

#### 6-mercaptopurine azathioprine

6-mercaptopurine azathioprine is the prodrug of 6-mercaptopurine (6-MP), and is non-enzymatically converted into 6-MP, where it has been shown that 6-MP is better tolerated than azathioprine [218]. A retrospective study of 22 patients with AIH who were switched to 6-MP therapy after treatment with the combination of azathioprine and prednisolone at two tertiary care institutions in Europe, a total of 15 of 20 patients with prior azathioprine intolerance (75%) responded to 6-MP treatment; eight of these patients had a CR and seven had partial remission, based on biochemical features. In these 15 patients, 6-MP was well tolerated, whereas the five remaining patients had to be switched to different immunosuppressive regimes because of 6-MP intolerance. The two patients with insufficient response to azathioprine treatment also showed no response to 6-MP, so 6-MP might be ineffective in patients with insufficient response to azathioprine [308]. A case report of three patients with AIH who could not tolerate azathioprine but tolerated 6-thioguanine 0.3 mg/kg daily well. All three patients improved clinically. Therapeutic drug monitoring was performed [309].

#### Infliximab

Infliximab is a recombinant humanized chimaeric antibody used for the treatment of ulcerative colitis, Crohn disease, rheumatoid arthritis, psoriatic arthritis/plaque psoriasis, and ankylosing spondylitis [310]. Eleven patients with difficult-to-treat AIH cohort treated with infliximab, showed decreasing in transaminases (mean AST prior treatment 475 U/L ± 466, mean AST during treatment 43 U/L ± 32) as well as in immunoglobulins (pretreatment mean IgG 24.8 mg/dL ± 10.1, mean IgG during treatment 17.38 mg/dL ± 6). With Infectious complications occurred in seven out of 11 patients including recurrent urinary tract infections, recurrent shingles, ocular herpes simplex infection, pneumonia, recurrent herpes labialis, bacterial abscess, ophthalmic shingles and allergic reaction [311]. A case report found an ankylosing spondylitis patient with elevation in transaminase levels. Transaminases including alanine and aspartate aminotransferases (ALT and AST) were found to be gradually increasing and finally became 500–600 IU/dL, excluded viral, metabolic and toxic causes for hepatitis. Serum anti-nuclear antibody (ANA) was 1/320 positive, and serum IgG was higher than normal (17.5 g/L). The liver biopsy showed an acute AIH with a predominantly lymphoplasmatic infiltration. Infliximab was ceased and immunosuppressive therapy was started (prednisolone 30 mg and azathioprine 50 mg). Serum AST and ALT became normal range at the second week of immunosuppressive drug therapy. Infliximab may be considered as rescue therapy in patients (5 mg/kg at day 0, weeks 2 and 6, and thereafter every 4–8 weeks depending on laboratory and clinical course) with difficult-to-treat AIH, albeit treatment may be associated with infectious complications, even themselves induce AIH sometimes [312].

#### Sirolimus

Sirolimus is a macrolide molecule acting by inhibiting the mammalian target of rapamycin, a protein that modulates the proliferation and survival of activated lymphocytes. The use and efficacy of sirolimus has been reported initially in the context of post-transplant AIH [313] and recently for refractory AIH in a non-transplant setting (median through level of 12.5 ng/mL): a sustained > 50% fall in ALT was achieved in 4/5 patients including normalization in two [314]. Main side effects of sirolimus include hyperlipidemia, proteinuria and edema, but it is relatively good safe. No strong recommendations can be drawn from such small sample sizes and it should be kept in mind that stronger immunosuppression is associated with severe infectious complications, especially in cirrhotic patients [311].

#### Other immunomodulatory therapy

Other agents have been used without strong evidence of efficacy, including cyclophosphamide (1–1.5 mg/kg/day) [315], methotrexate (7.5 mg/week) [316], tioguanine [317] and rituximab (1000 mg 2 weeks apart) [318].

### Guidance

If the first-line treatment does not respond, or if the response is achieved but the adverse reaction is not tolerated, second-line treatment can be chosen.

### Transplantation for AIH

AIH can lead to acute liver failure and end-stage liver disease. Liver transplantation (LT) is an effective therapy for AIH patients with decompensated cirrhosis whose MELD score ≥ 15 and in patients who present with acute liver failure. Although data regarding LT in patients with AIH are limited, the overall survival rate in AIH appears to be excellent in AIH: the 5- and 10-year recipient survival rates are 76–79% and 67–75%, respectively, which are better than for most other indications for LT [319–321] (Table 6).

**Table 6:** Patient and graft survival at 5 and 10 years after LT

Region	*n*	Patient survival		Graft survival	
		5 years	10 years	5 years	10 years
Europe	1892	76	67	69	59
Japan	104	79	75	NA	NA

Registry data from Europe [319], USA [320] and Japan [321]

On the other hand, recurrence of AIH in the graft after LT is common, and it is very challenging to determine the incidence of recurrent AIH, which is reported to be in the range of 7–42% [322–332] (Table 7). The inconsistency among studies is likely due to differences in diagnostic criteria, histological analysis (protocol or event-driven biopsy), small sample size in each study (no study with more than 100 patients enrolled), and follow-up time [333, 334]. The rate of recurrence increases as the follow-up time increases after LT [323, 329, 330]. Neither the revised criteria, [335] nor the simplified criteria, [174] are validated for the diagnosis of recurrent AIH.

**Table 7:** Incidence and risk factors of recurrence of AIH after LT

Center sites	Time period	Year	*n*	Incidence	Time to recurrence (years)^a^
Spain [330]	1988–1996	1998	27	9 (33%)	2.6 ± 1.5
Birmingham, UK [327]	NA	1999	47	13 (28%)	2.4 (0.5–5.3)
Paris, France [331]	1985–1992	1999	15	3 (20%)	1.6 (1–2.5)
New York, USA [332]	1988–1995	2000	24	6 (25%)	1.3 ± 0.2
Boston, USA [322]	1983–1998	2000	12	5 (42%)	NA
Rochester, USA [325]	1985–1998	2001	41	7 (17%)	4.6 ± 1
Dallas, USA [328]	1984–1998	2002	55	11 (20%)	NA
Paris, France [324]	1985–1992	2003	17	7 (41%)	2.5 ± 1.7
Colorado, USA [323]	1988–2006	2008	66	23 (34.8%)	4.3
Alberta, Canada [329]	NA	2009	46	11 (24%)	4 ± 1.3
Birmingham, UK [326]	1999–2014	2016	69	5 (7%)	3.8 (1.5–7.3)

*NA* not available

^a^Time to recurrence was shown as median (range), or mean ± SD

A number of factors are reported to be associated with the recurrence of AIH, including the severity of pre-transplant AIH [322, 329] and withdrawal of corticosteroids [327, 330]. HLA locus mismatching was identified as a risk factor for recurrence [336] in one study but not in others [327–329, 332]. A recent study from the UK demonstrated that the 5- and 10-year recurrence rates after LT were 6 and 11%, respectively, in their cohort consisting of 69 patients with AIH, in which 87% of patients were under long-term maintenance treatment with corticosteroids after LT [326]. Compared to the recurrence rate of 27% in their previous report in 1999 in patients without long-term corticosteroid therapy [327], the authors concluded that long-term corticosteroid use in combination with immunosuppressive agents was associated with a lower frequency of recurrence.

In general, progressing to cirrhosis and graft failure requiring re-transplantation is uncommon, even when AIH recurs in the graft [333]. However, the mechanisms that cause recurrent AIH after LT remains unclear. Furthermore, there are also substantial differences between adults and pediatric patients with de novo AIH, which substantiates the need for more precise diagnostic guidelines in this area [337, 338]. When recurrence occurs in the graft, the strength of immunosuppression should be reinforced with re-administration or dosing-up of corticosteroids, or the addition of other immunosuppressive agents.

Another issue after LT is development of de novo AIH. De novo AIH has an incidence between 0.5 and 3.4% in adults with reported time to development ranging from 0.3 to 7 years post-LT [339]. De novo AIH may occur more commonly in children and the incidence between 0.5 and 11%, and time to development is ranging from 1.2 to 6.9 years [340, 341]. Treatment for de novo AIH is similar to standard treatment for recurrent AIH after LT. Most cases can be treated effectively, but others may progress to graft failure and require re-transplantation [342].

### Guidance

1. Liver transplantation should be considered in patients with decompensated AIH who do not respond to or are not suitable for drug therapies.
2. Liver transplantation should be considered in AIH patients presenting as acute liver failure if recovery is impossible to achieve.
3. Treatment of AIH following liver transplantation (recurrent or de novo) should follow the standard management of AIH.

### Treating AIH in the context of liver co-morbidity

#### AIH-PBC overlap syndrome

The low prevalence of AIH-PBC overlap syndrome has made controlled treatment studies not feasible so that the treatment recommendations rely on retrospective studies and the treatment of either PBC or AIH. Treatment with UDCA is recommended for PBC [343]. For AIH, immunosuppressive treatment (with either corticosteroids alone or in combination with AZA) is recommended when meet the therapeutic indications. Although UDCA therapy alone may induce biochemical responses in some patients with AIH-PBC overlap, most patients may require a combination of UDCA and immunosuppressive therapy to obtain a CR [344].

Seventeen strictly defined patients with AIH-PBC overlap received either UDCA alone or in combination with immunosuppressive therapy were followed up for an average of 7.5 years [344]. The overall fibrosis progression in noncirrhotic patients occurred more frequently under UDCA monotherapy (4 of 8) than under combined therapy (0 of 6), suggesting that combined therapy may be the best option [344]. In clinical practice, it is very important to identify patients who would benefit from UDCA alone and those patients who require immunosuppression in addition to UDCA. An international multi-centre study evalauted data of 88 patients with AIH-PBC overlap [345]. Patients with severe activity of AIH were less likely to respond UDCA montherapy and oftenly required additional immünsupression while UDCA alone induced biocemical remission in the majority of patients with modarete active AIH. These results suggest that initial therapy regimen can be determined according to histologic findings for patients with AIH-PBC overlap. Second-line immunosuppressive agents (CsA, tacrolimus, and MMF) are effective in controlling disease activity in patients who do not respond to conventional immunosuppression.

Patients with AIH or PBC who exhibit features that are suspicious for overlap syndrome, but do not meet the criteria, should be treated according to the clinically predominant disease [346]. Immunosuppressive therapy is indicated for AIH-predominant patients, and UDCA is indicated for PBC-predominant patients.

#### AIH-PSC overlap syndrome

Unlike the classical PSC, patients with AIH-PSC overlap seem to derive some benefit from UDCA and immunosuppressive agents, and the survival rates are apparently better than in classical PSC, but with a poorer outcome than classical AIH and AIH-PBC overlap [347, 348]. In a prospective Italian study, 41 consecutive PSC patients (7 fulfilled the criteria for AIH-PSC overlap syndrome) were treated either with immunosuppressive agents plus UDCA in those with AIH-PSC overlap or with UDCA in those with classical PSC [347]. In patients with AIH-PSC overlap, a 5-year treatment with immunosuppressive agents plus UDCA was significantly effective in improving AST, drop in serum ALT, gamma-glutamyl transferase (GGT), and ALP was also obtained, but without reaching a statistical significance. A biochemical response to immunosuppressive therapy in AIH-PSC overlap patients has also been reported in another study from Sweden and in a study in children [282, 349]. Patients with AIH-PSC overlap syndrome be treated with UDCA and immunosuppressive therapy but emphasizes that this is not evidence based.

Suggested treatment and outcomes of AIH and its overlap syndromes shown in Table 8.

**Table 8:** Suggested treatment and outcomes of AIH and its overlap syndromes

	Treatment	Outcomes
AIH-PBC overlap	Immunosuppressive therapy and UDCA, 13–15 mg/kg/day In patients with mild active AIH, can start with UDCA only and to add immunosuppressive drugs if insufficient response in 3 months	Biochemical response achieved in most patients Overall prognosis: worse than classical PBC, and may be slightly worse than AIH alone
AIH-PSC overlap	Immunosuppressive drugs with or without UDCA, 13–15 mg/kg/day	Biochemical response is variable Overall prognosis: most progress to cirrhosis after 10 years, better than classical PSC, and worse than AIH alone

#### Drug-induced AIH (DI-AIH)

**Fig. 3:**
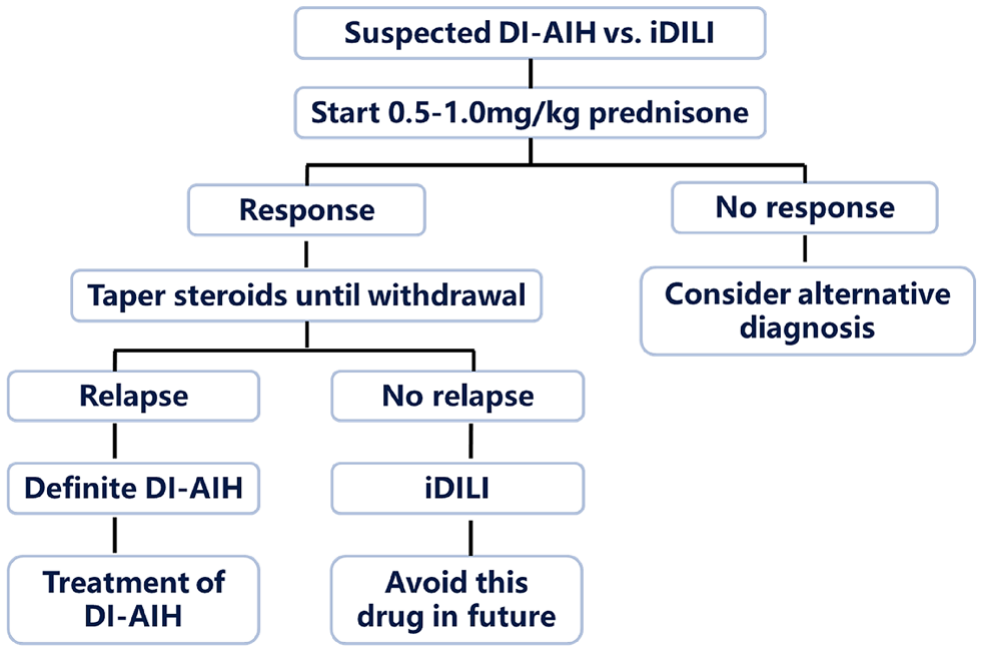
Steroid treatment and observation in patients with DI-AIH

Drug-induced AIH (no reports for PBC or PSC) is a poorly defined and under-reported liver disorder, and, probably, an underestimated liver disease. A small number of drug-induced liver injury (DILI) cases exhibit features typical of AIH. To differentiate between true AIH triggered by drugs (DI-AIH) and immune mediated DILI (iDILI) still remains a challenge [350]. Severe DI-AIH usually responds to high doses of steroids in the same way as severe AIH, if treatment is started without delay. Sometimes, only the follow-up can differentiate between AIH and DILI: steroid treatment can be discontinued without relapse in DILI, whereas in genuine AIH relapse will occur universally, if immunosuppression is stopped within a few months (Fig. 3). A trial of steroid treatment and close observation upon steroid tapering and possible withdrawal are therefore recommended for uncertain cases [12, 351].

The most common drugs causing DI-AIH were furantoin and minocycline, as well as non-steroidal anti-inflammatory drugs, interferon, dihydrazine-bendazine, halothane, phenol-butyl, tenic acid, methyldopa, statins, ephedra, etc. With the wide application of immunocheckpoint inhibitors (ICIs) in clinic, more and more attention has been paid to some adverse reactions caused by immunotherapy, immune-related adverse events (irAEs), among which immunological hepatitis is one of the potential serious complications. Treatment for patients with immune hepatitis includes discontinuation of ICIs, first-line glucocorticoid therapy, and second-line immunosuppressant therapy (e.g., mycophonate, tacrolimus, etc.) [352].

#### Chronic viral hepatitis and AIH

Patients with AIH and HBV infection (HBsAg positive) should receive Entecavir (ETV) or Tenofovir disoproxil fumarate (TDF) or Tenofovir alafenamide (TAF) as treatment or prophylaxis, and then immunosuppressive therapy was started. Once started, anti-HBV prophylaxis should continue during immunosuppressive therapy and for at least 6 months after completion of immunosuppressive therapy. Patients should be monitored in HBV DNA levels for up to 12 months after cessation of anti-HBV therapy [353].

Patients with AIH and HCV infection (HCV RNA positive) should be treated with interferon-free, direct-acting antiviral agents (DAA)-based anti-HCV combinations, and then or at the same time immunosuppressive therapy was started. The selection of direct-acting antiviral agents can according to the recommendations for HCV infection alone, in combination with immunosuppressive therapy, take into account possible drug–drug interactions [354].

#### Non-alcoholic fatty liver disease (NAFLD) and AIH

AIH and coincident NAFLD are not a rare condition. Patients with coincident AIH and nonalcoholic steatohepatitis (NASH) are more likely to present with cirrhosis and more likely to develop an adverse clinical outcome with poorer survival compared to AIH alone [355]. Histological information is useful for determining the diagnosis and selecting the treatment agents in patients with NASH and AIH [356]. Corticosteroid therapy is necessary when the focal necrosis is confirmed through liver histology. Corticosteroids may exaggerate fat deposition in hepatocytes, worsen NASH, and reduce the inflammatory activity of AIH, therefore, the efficacy of corticosteroids in AIH and NASH may be decreased. AIH and coincident NAFLD patients must be closely monitored and corticosteroids must be replaced by other immunosuppressive drugs if standard corticosteroids therapy fails to achieve remission [356].

#### HIV infection and AIH

Standard immunosuppressive therapy for AIH is effective, but sometimes associated with life-threatening infections. Individualized treatment of AIH in HIV-infected patients should be carried out after careful consideration of potential risks and possible benefits [357].

### Guidance

1. In patients with AIH-PBC overlap syndrome, combined therapy with UDCA and immunosuppressants is recommended.
2. In patients with AIH-PSC overlap syndrome, addition of UDCA to immunosuppressant can be considered.
3. In patients with dominant AIH features, can start with immunosuppressants only and then add UDCA if response is insufficient.
4. Patients with AIH and HBV infection should receive ETV or TDF or TAF as treatment or prophylaxis, and then immunosuppressive therapy was started.
5. Patients with AIH and HCV infection should be treated with interferon-free, DAA-based anti-HCV combinations, and then or at the same time immunosuppressive therapy was started.

## Natural history, prognosis and survival

AIH is a chronic and progressive hepatitis, and could promptly progress to cirrhosis if untreated. Several randomized controlled studies were conducted in the 1960s and 1970s [198–202], and a systematic review of these randomized trials was published in 2010 [203]. Among them, Cook et al. conducted a randomized, controlled, prospective clinical trial in patients with active chronic hepatitis between prednisolone monotherapy (15 mg/day) and placebo. After 72 months of observation, three out of 22 patients treated with prednisolone (14%) and 15 out of 27 patients with placebo (56%) had died [198]. The mean value of AST, serum bilirubin and albumin in placebo group was 118 IU/L, 3.8 mg/dL and 3.0 g/dL, respectively, indicating that AIH patients with decompensated cirrhosis have a high mortality, 56% at 6 months, while intervention with prednisolone decreased mortality to 14%. Another placebo-controlled trial was performed by Soloway et al., in which 17 patients with chronic active hepatitis served as a placebo control [200], and again high mortality (41% died up to 3.5 years) was noted. Even without cirrhosis at presentation, AIH patients with bridging necrosis or multilobular necrosis are likely to progress to cirrhosis in a few years if untreated, and long-term prognosis is poor [204, 205].

On the other hand, the natural history of asymptomatic AIH patients with mild laboratory and histological abnormalities remains largely unknown [66]. Feld et al. demonstrated that AIH patients with asymptomatic at presentation, who had lower serum aminotransferase, bilirubin and IgG, had a good prognosis with 80.0% of 10-year survival even though half of these patients received no treatment, while patients with cirrhosis at baseline exhibited poorer 10-year survival (61.9%) [52]. Nevertheless, 80% of the 10-year survival does not appear to be excellent currently since 10-year survival of patients with AIH exceeds 90% [206]. Furthermore, we have not had biomarkers or histological findings to identify “safe” AIH patients who do not require corticosteroids therapy. It should be noted that the mild AIH can progress to severe fibrosis, leading to poor outcomes.

### Hepatocellular carcinoma

Although occurrence of hepatocellular carcinoma (HCC) was considered to be a rare event in patients with AIH, recent clinical studies regarding natural history of AIH clearly indicate the importance of HCC in AIH as comorbidity, along with improved survival of patients over time. A systematic review of 25 studies demonstrated the pooled incidence rate for HCC in patients with AIH was 3.06 per 1000 patient-years (95% CI 2.22–4.23) [358]. Another systematic review suggested the development of HCC was not altered before and after the discovery of HCV [359].

In Table 9, the reported case series and incidence of HCC in patients with AIH are summarized. While study designs, detection methods of HCC, and proportion of cirrhosis at baseline greatly varied, the incidence of HCC ranged around 3–5 per 1000 person-years except for two studies [6, 70, 360–364], which should not be underestimated even though substantially lower than those in liver diseases due to HBV or HCV infection [358]. Cirrhosis at presentation or during clinical course is definitely associated with development of HCC in patients with AIH [70, 360–362, 365]. Repeated relapse and elevated ALT also contribute to development of HCC [206, 360, 362]. Other risk factors may include old age, disease duration [366], male gender, immunosuppressive treatment for > 3 years [362]. Taken together, regular HCC surveillance is justified and should be scheduled in patients with AIH, especially in cirrhotic patients.

**Table 9:** Reported HCC cases in patients with AIH

Area	Country		No. of AIH	No. of HCC	Mean follow-up (months)	Incidence*	% cirrhosis at baseline	Year	References
Asia–Pacific	Japan	Migita	193	7	96.0	4.53	10.9	2012	[361]
	Japan	Hino-Arinaga	180	6	80.2	5.00	18.9	2012	[360]
	South Korea	Kim	4085	31	60	1.52	32.3	2017	[6]
Europe and America	UK	Yeoman	243	15	149.6	4.95	50.2	2008	[70]
	USA	Montano-Loza	227	9	134.0	3.55	43.2	2008	[362]
	Germany	Teufel	278	0	57.6	0	32	2009	[363]
	USA	Wong	322	6	75.0	4.59	1.6	2011	[364]

*HCC per 1000 person-years

In the largest case series from Japan comprising 127 patients of HCC in patients with AIH [367], 78% of patients were at cirrhotic stage, and the proportion of those at Child–Pugh A/B/C was 61.8, 30.9 and 7.3%, respectively. The maximum tumor size was 4.3 cm in average (range 1.0–30.0), and regarding clinical stage, I/II/III/IV was 20.1, 47.6, 23.4 and 8.9%, indicating that diagnosis of HCC was made at the earlier stages. Treatment protocols were similar to those in patients with other etiologies, and cumulative survival rates were 65.8 and 56.4% at 3 and 5 years, respectively.

### Guidance

- (1) Regular surveillance of HCC is advisable in patients with AIH.
- (2) Cirrhosis, repeated relapse, and old age are significantly associated with development of HCC, and intensive surveillance of HCC is strongly recommended in patients with these risk factors.
- (3) Treatment for HCC should be similarly conducted as other etiologies.

## References

[1] Yang F, Wang Q, Bian Z, Ren LL, Jia J, Ma X (2015). Autoimmune hepatitis: east meets west. J Gastroenterol Hepatol.

[2] Lv T, Li M, Zeng N, Zhang J, Li S, Chen S (2019). Systematic review and meta-analysis on the incidence and prevalence of autoimmune hepatitis in Asian, European, and American population. J Gastroenterol Hepatol.

[3] Wong RJ, Gish R, Frederick T, Bzowej N, Frenette C (2012). The impact of race/ethnicity on the clinical epidemiology of autoimmune hepatitis. J Clin Gastroenterol.

[4] Tanaka A, Ma X, Yokosuka O, Weltman M, You H, Amarapurkar DN (2016). Autoimmune liver diseases in the Asia-Pacific region: proceedings of APASL symposium on AIH and PBC 2016. Hepatol Int.

[5] Tanaka A, Mori M, Matsumoto K, Ohira H, Tazuma S, Takikawa H (2019). Increase trend in the prevalence and male-to-female ratio of primary biliary cholangitis, autoimmune hepatitis, and primary sclerosing cholangitis in Japan. Hepatol Res.

[6] Kim BH, Choi HY, Ki M, Kim KA, Jang ES, Jeong SH (2017). Population-based prevalence, incidence, and disease burden of autoimmune hepatitis in south Korea. PLoS ONE.

[7] Ngu JH, Bechly K, Chapman BA, Burt MJ, Barclay ML, Gearry RB (2010). Population-based epidemiology study of autoimmune hepatitis: a disease of older women?. J Gastroenterol Hepatol.

[8] Valgeirsson KB, Hreinsson JP, Bjornsson ES (2019). Increased incidence of autoimmune hepatitis is associated with wider use of biological drugs. Liver Int.

[9] Mells GF, Kaser A, Karlsen TH (2013). Novel insights into autoimmune liver diseases provided by genome-wide association studies. J Autoimmun.

[10] Werner M, Prytz H, Ohlsson B, Almer S, Bjornsson E, Bergquist A (2008). Epidemiology and the initial presentation of autoimmune hepatitis in Sweden: a nationwide study. Scand J Gastroenterol.

[11] Wang Q, Yang F, Miao Q, Krawitt EL, Gershwin ME, Ma X (2016). The clinical phenotypes of autoimmune hepatitis: a comprehensive review. J Autoimmun.

[12] EASL (2015). EASL clinical practice guidelines: autoimmune hepatitis. J Hepatol.

[13] Floreani A, Restrepo-Jimenez P, Secchi MF, De Martin S, Leung PSC, Krawitt E (2018). Etiopathogenesis of autoimmune hepatitis. J Autoimmun.

[14] Umemura T, Katsuyama Y, Yoshizawa K, Kimura T, Joshita S, Komatsu M (2014). Human leukocyte antigen class II haplotypes affect clinical characteristics and progression of type 1 autoimmune hepatitis in Japan. PLoS ONE.

[15] Oka S, Furukawa H, Yasunami M, Kawasaki A, Nakamura H, Nakamura M (2017). HLA-DRB1 and DQB1 alleles in Japanese type 1 autoimmune hepatitis: the predisposing role of the DR4/DR8 heterozygous genotype. PLoS ONE.

[16] Amarapurkar DN, Patel ND, Amarapurkar AD, Kankonkar SR (2003). HLA genotyping in type-I autoimmune hepatitis in western India. J Assoc Physicians India.

[17] Kaur N, Minz RW, Anand S, Saikia B, Aggarwal R, Das A (2014). HLA DRB1 alleles discriminate the manifestation of autoimmune hepatitis as type 1 or type 2 in north Indian population. J Clin Exp Hepatol.

[18] Hiraide A, Imazeki F, Yokosuka O, Kanda T, Kojima H, Fukai K (2005). Fas polymorphisms influence susceptibility to autoimmune hepatitis. Am J Gastroenterol.

[19] Umemura T, Joshita S, Yamazaki T, Komatsu M, Katsuyama Y, Yoshizawa K (2016). Genetic association of PTPN22 polymorphisms with autoimmune hepatitis and primary biliary cholangitis in Japan. Sci Rep.

[20] Fan L, Tu X, Zhu Y, Zhou L, Pfeiffer T, Feltens R (2005). Genetic association of vitamin D receptor polymorphisms with autoimmune hepatitis and primary biliary cirrhosis in the Chinese. J Gastroenterol Hepatol.

[21] de Boer YS, van Gerven NM, Zwiers A, Verwer BJ, van Hoek B, van Erpecum KJ (2014). Genome-wide association study identifies variants associated with autoimmune hepatitis type 1. Gastroenterology.

[22] Webb GJ, Hirschfield GM (2016). Using GWAS to identify genetic predisposition in hepatic autoimmunity. J Autoimmun.

[23] Seki T, Ota M, Furuta S, Fukushima H, Kondo T, Hino K (1992). HLA class II molecules and autoimmune hepatitis susceptibility in Japanese patients. Gastroenterology.

[24] Strettell MD, Donaldson PT, Thomson LJ, Santrach PJ, Moore SB, Czaja AJ (1997). Allelic basis for HLA-encoded susceptibility to type 1 autoimmune hepatitis. Gastroenterology.

[25] Czaja AJ, Strettell MD, Thomson LJ, Santrach PJ, Moore SB, Donaldson PT (1997). Associations between alleles of the major histocompatibility complex and type 1 autoimmune hepatitis. Hepatology.

[26] Vazquez-Garcia MN, Alaez C, Olivo A, Debaz H, Perez-Luque E, Burguete A (1998). MHC class II sequences of susceptibility and protection in Mexicans with autoimmune hepatitis. J Hepatol.

[27] Bittencourt PL, Goldberg AC, Cancado EL, Porta G, Carrilho FJ, Farias AQ (1999). Genetic heterogeneity in susceptibility to autoimmune hepatitis types 1 and 2. Am J Gastroenterol.

[28] Muratori P, Czaja AJ, Muratori L, Pappas G, Maccariello S, Cassani F (2005). Genetic distinctions between autoimmune hepatitis in Italy and north America. World J Gastroenterol.

[29] Teufel A, Worns M, Weinmann A, Centner C, Piendl A, Lohse AW (2006). Genetic association of autoimmune hepatitis and human leucocyte antigen in German patients. World J Gastroenterol.

[30] Al-Chalabi T, Boccato S, Portmann BC, McFarlane IG, Heneghan MA (2006). Autoimmune hepatitis (AIH) in the elderly: a systematic retrospective analysis of a large group of consecutive patients with definite AIH followed at a tertiary referral centre. J Hepatol.

[31] Djilali-Saiah I, Fakhfakh A, Louafi H, Caillat-Zucman S, Debray D, Alvarez F (2006). HLA class II influences humoral autoimmunity in patients with type 2 autoimmune hepatitis. J Hepatol.

[32] Mdel PF, Machado IV, Gil G, Fernandez-Mestre M, Dagher L, Leon RV (2007). Genetic contribution of major histocompatibility complex class II region to type 1 autoimmune hepatitis susceptibility in Venezuela. Liver Int.

[33] Lim YS, Oh HB, Choi SE, Kwon OJ, Heo YS, Lee HC (2008). Susceptibility to type 1 autoimmune hepatitis is associated with shared amino acid sequences at positions 70–74 of the HLA-DRB1 molecule. J Hepatol.

[34] Czaja AJ (2015). Transitioning from idiopathic to explainable autoimmune hepatitis. Dig Dis Sci.

[35] Christen U, Hintermann E (2014). Pathogen infection as a possible cause for autoimmune hepatitis. Int Rev Immunol.

[36] Gatselis NK, Zachou K, Koukoulis GK, Dalekos GN (2015). Autoimmune hepatitis, one disease with many faces: etiopathogenetic, clinico-laboratory and histological characteristics. World J Gastroenterol.

[37] Efe C, Kav T, Aydin C, Cengiz M, Imga NN, Purnak T (2014). Low serum vitamin D levels are associated with severe histological features and poor response to therapy in patients with autoimmune hepatitis. Dig Dis Sci.

[38] Albert LJ, Inman RD (1999). Molecular mimicry and autoimmunity. N Engl J Med.

[39] Gleeson D, Heneghan MA, British Society of Gastroenterology (2011). British Society of Gastroenterology (BSG) guidelines for management of autoimmune hepatitis. Gut.

[40] Czaja AJ (2011). Drug-induced autoimmune-like hepatitis. Dig Dis Sci.

[41] Czaja AJ (2007). Autoimmune hepatitis. Part A: pathogenesis. Expert Rev Gastroenterol Hepatol.

[42] Mieli-Vergani G, Vergani D, Czaja AJ, Manns MP, Krawitt EL, Vierling JM (2018). Autoimmune hepatitis. Nat Rev Dis Primers.

[43] Lin R, Zhou L, Zhang J, Wang B (2015). Abnormal intestinal permeability and microbiota in patients with autoimmune hepatitis. Int J Clin Exp Pathol.

[44] Vieira SM, Hiltensperger M, Kumar V, Zegarra-Ruiz D, Dehner C, Khan N (2018). Translocation of a gut pathobiont drives autoimmunity in mice and humans. Science.

[45] Wei Y, Li Y, Yan L, Sun C, Miao Q, Wang Q (2020). Alterations of gut microbiome in autoimmune hepatitis. Gut.

[46] Longhi MS, Ma Y, Bogdanos DP, Cheeseman P, Mieli-Vergani G, Vergani D (2004). Impairment of CD4(+)CD25(+) regulatory T-cells in autoimmune liver disease. J Hepatol.

[47] Longhi MS, Ma Y, Mitry RR, Bogdanos DP, Heneghan M, Cheeseman P (2005). Effect of CD4+ CD25+ regulatory T-cells on CD8 T-cell function in patients with autoimmune hepatitis. J Autoimmun.

[48] Longhi MS, Hussain MJ, Mitry RR, Arora SK, Mieli-Vergani G, Vergani D (2006). Functional study of CD4+CD25+ regulatory T cells in health and autoimmune hepatitis. J Immunol.

[49] Ferri S, Longhi MS, De Molo C, Lalanne C, Muratori P, Granito A (2010). A multifaceted imbalance of T cells with regulatory function characterizes type 1 autoimmune hepatitis. Hepatology.

[50] Zhao L, Tang Y, You Z, Wang Q, Liang S, Han X (2011). Interleukin-17 contributes to the pathogenesis of autoimmune hepatitis through inducing hepatic interleukin-6 expression. PLoS ONE.

[51] Zhang H, Liu Y, Bian Z, Huang S, Han X, You Z (2014). The critical role of myeloid-derived suppressor cells and FXR activation in immune-mediated liver injury. J Autoimmun.

[52] Feld JJ, Dinh H, Arenovich T, Marcus VA, Wanless IR, Heathcote EJ (2005). Autoimmune hepatitis: effect of symptoms and cirrhosis on natural history and outcome. Hepatology.

[53] Kogan J, Safadi R, Ashur Y, Shouval D, Ilan Y (2002). Prognosis of symptomatic versus asymptomatic autoimmune hepatitis: a study of 68 patients. J Clin Gastroenterol.

[54] Czaja AJ (2005). Diverse manifestations and evolving treatments of autoimmune hepatitis. Minerva Gastroenterol Dietol.

[55] Krawitt EL (2006). Autoimmune hepatitis. N Engl J Med.

[56] Czaja AJ, Manns MP (2010). Advances in the diagnosis, pathogenesis, and management of autoimmune hepatitis. Gastroenterology.

[57] Liberal R, Grant CR, Mieli-Vergani G, Vergani D (2013). Autoimmune hepatitis: a comprehensive review. J Autoimmun.

[58] Yamamoto K, Miyake Y, Ohira H, Suzuki Y, Zeniya M, Onji M (2013). Prognosis of autoimmune hepatitis showing acute presentation. Hepatol Res.

[59] Joshita S, Yoshizawa K, Umemura T, Ohira H, Takahashi A, Harada K (2018). Clinical features of autoimmune hepatitis with acute presentation: a Japanese nationwide survey. J Gastroenterol.

[60] Abe M, Mashiba T, Zeniya M, Yamamoto K, Onji M, Tsubouchi H (2011). Present status of autoimmune hepatitis in Japan: a nationwide survey. J Gastroenterol.

[61] Miyake Y, Iwasaki Y, Kobashi H, Yasunaka T, Ikeda F, Takaki A (2010). Autoimmune hepatitis with acute presentation in Japan. Dig Liver Dis.

[62] Anand L, Choudhury A, Bihari C, Sharma BC, Kumar M, Maiwall R (2018). Flare of autoimmune hepatitis causing acute on chronic liver failure (ACLF): diagnosis and response to corticosteroid therapy. Hepatology.

[63] Iwai M, Jo M, Ishii M, Mori T, Harada Y (2008). Comparison of clinical features and liver histology in acute and chronic autoimmune hepatitis. Hepatol Res.

[64] Weiler-Normann C, Lohse AW (2014). Acute autoimmune hepatitis: many open questions. J Hepatol.

[65] Dalekos GN, Gatselis NK, Zachou K (2019). Acute severe autoimmune hepatitis: corticosteroids or liver transplantation?. Liver Transpl.

[66] Manns MP, Czaja AJ, Gorham JD, Krawitt EL, Mieli-Vergani G, Vergani D (2010). Diagnosis and management of autoimmune hepatitis. Hepatology.

[67] Miyake Y, Iwasaki Y, Terada R, Nagano T, Kobashi H, Sakaguchi K (2008). A model for estimating cirrhosis in patients with type 1 autoimmune hepatitis. Hepatol Res.

[68] Abe K, Katsushima F, Kanno Y, Takahashi A, Yokokawa J, Ohira H (2012). Clinical features of cirrhosis in Japanese patients with type I autoimmune hepatitis. Intern Med.

[69] Zachou K, Muratori P, Koukoulis GK, Granito A, Gatselis N, Fabbri A (2013). Review article: autoimmune hepatitis—current management and challenges. Aliment Pharmacol Ther.

[70] Yeoman AD, Al-Chalabi T, Karani JB, Quaglia A, Devlin J, Mieli-Vergani G (2008). Evaluation of risk factors in the development of hepatocellular carcinoma in autoimmune hepatitis: implications for follow-up and screening. Hepatology.

[71] Johnson PJ, McFarlane IG, OBotP Convenors (1993). Meeting report: International autoimmune hepatitis group. Hepatology.

[72] Alvarez F, Berg PA, Bianchi FB, Bianchi L, Burroughs AK, Cancado EL (1999). International autoimmune hepatitis group report: review of criteria for diagnosis of autoimmune hepatitis. J Hepatol.

[73] Hennes EM, Zeniya M, Czaja AJ, Parés A, Dalekos GN, Krawitt EL (2008). Simplified criteria for the diagnosis of autoimmune hepatitis. Hepatology.

[74] The French METAVIR Cooperative Study Group (1994). Intraobserver and interobserver variations in liver biopsy interpretation in patients with chronic hepatitis C. Hepatology.

[75] Rastogi A, Kumar A, Sakhuja P, Bihari C, Gondal R, Hissar S (2011). Liver histology as predictor of outcome in patients with acute-on-chronic liver failure (ACLF). Virchows Arch.

[76] Kirstein MM, Metzler F, Geiger E, Heinrich E, Hallensleben M, Manns MP (2015). Prediction of short- and long-term outcome in patients with autoimmune hepatitis. Hepatology.

[77] Muratori P, Lalanne C, Bianchi G, Lenzi M, Muratori L (2016). Predictive factors of poor response to therapy in autoimmune hepatitis. Dig Liver Dis.

[78] Czaja AJ, Carpenter HA (2003). Histological features associated with relapse after corticosteroid withdrawal in type 1 autoimmune hepatitis. Liver Int.

[79] Dhaliwal HK, Hoeroldt BS, Dube AK, McFarlane E, Underwood JC, Karajeh MA (2015). Long-term prognostic significance of persisting histological activity despite biochemical remission in autoimmune hepatitis. Am J Gastroenterol.

[80] FF van den Brand, KS van der Veen, YS de Boer, NM van Gerven, BI Lissenberg-Witte, U Beuers et al. Increased mortality among patients with vs without cirrhosis and autoimmune hepatitis*.* Clin Gastroenterol Hepatol. 2019;17(5):940–947.e2. 10.1016/j.cgh.2018.09.046. 10.1016/j.cgh.2018.09.046 30291909

[81] Puustinen L, Boyd S, Mustonen H, Arkkila P, Arola J, Farkkila M (2017). Prognostic value of clinical variables and liver histology for development of fibrosis and cirrhosis in autoimmune hepatitis. Scand J Gastroenterol.

[82] Harada K, Hiep NC, Ohira H (2017). Challenges and difficulties in pathological diagnosis of autoimmune hepatitis. Hepatol Res.

[83] Stirnimann G, Ebadi M, Czaja AJ, Montano-Loza AJ (2019). Recurrent and de novo autoimmune hepatitis. Liver Transpl.

[84] Chapin CA, Mohammad S, Bass LM, Taylor SA, Kelly S, Alonso EM (2018). Liver biopsy can be safely performed in pediatric acute liver failure to aid in diagnosis and management. J Pediatr Gastroenterol Nutr.

[85] Stift J, Semmler G, Walzel C, Mandorfer M, Schwarzer R, Schwabl P (2019). Transjugular aspiration liver biopsy performed by hepatologists trained in HVPG measurements is safe and provides important diagnostic information. Dig Liver Dis.

[86] Cholongitas E, Burroughs AK (2012). Liver: transjugular liver biopsy yields high-quality samples. Nat Rev Gastroenterol Hepatol.

[87] Kalambokis G, Manousou P, Vibhakorn S, Marelli L, Cholongitas E, Senzolo M (2007). Transjugular liver biopsy–indications, adequacy, quality of specimens, and complications–a systematic review. J Hepatol.

[88] van Leeuwen DJ, Alves V, Balabaud C, Bhathal PS, Bioulac-Sage P, Colombari R (2018). Acute-on-chronic liver failure 2018: a need for (urgent) liver biopsy?. Expert Rev Gastroenterol Hepatol.

[89] Denzer U, Arnoldy A, Kanzler S, Galle PR, Dienes HP, Lohse AW (2007). Prospective randomized comparison of minilaparoscopy and percutaneous liver biopsy: diagnosis of cirrhosis and complications. J Clin Gastroenterol.

[90] Denzer U, Helmreich-Becker I, Galle PR, Lohse AW (2003). Liver assessment and biopsy in patients with marked coagulopathy: value of mini-laparoscopy and control of bleeding. Am J Gastroenterol.

[91] Helmreich-Becker I, zum Buschenfelde KHM, Lohse AW (1998). Safety and feasibility of a new minimally invasive diagnostic laparoscopy technique. Endoscopy.

[92] Zeng J, Huang Z-P, Zheng J, Wu T, Zheng R-Q (2017). Non-invasive assessment of liver fibrosis using two-dimensional shear wave elastography in patients with autoimmune liver diseases. World J Gastroenterol.

[93] Hartl J, Ehlken H, Sebode M, Peiseler M, Krech T, Zenouzi R (2018). Usefulness of biochemical remission and transient elastography in monitoring disease course in autoimmune hepatitis. J Hepatol.

[94] J Hartl, H Ehlken, M Sebode, M Peiseler, T Krech, R Zenouzi et al. (2017) Usefulness of biochemical remission and transient elastography in monitoring disease course in autoimmune hepatitis*.* J Hepatol. 2018:68(4):754–763. 10.1016/j.jhep.2017.11.020. 10.1016/j.jhep.2017.11.020 29180000

[95] Yada N, Sakurai T, Minami T, Arizumi T, Takita M, Hagiwara S (2017). Influence of liver inflammation on liver stiffness measurement in patients with autoimmune hepatitis evaluation by combinational elastography. Oncology.

[96] Guo L, Zheng L, Hu L, Zhou H, Yu L, Liang W (2017). Transient elastography (Fibroscan) performs better than non-invasive markers in assessing liver fibrosis and cirrhosis in autoimmune hepatitis patients. Med Sci Monit.

[97] Xu Q, Sheng L, Bao H, Chen X, Guo C, Li H (2017). Evaluation of transient elastography in assessing liver fibrosis in patients with autoimmune hepatitis. J Gastroenterol Hepatol.

[98] Hartl J, Denzer U, Ehlken H, Zenouzi R, Peiseler M, Sebode M (2016). Transient elastography in autoimmune hepatitis: timing determines the impact of inflammation and fibrosis. J Hepatol.

[99] Czaja AJ, Carpenter HA (1993). Sensitivity, specificity, and predictability of biopsy interpretations in chronic hepatitis. Gastroenterology.

[100] Mieli-Vergani G, Vergani D, Baumann U, Czubkowski P, Debray D, Dezsofi A (2018). Diagnosis and management of pediatric autoimmune liver disease: ESPGHAN hepatology committee position statement. J Pediatr Gastroenterol Nutr.

[101] Canh HN, Harada K, Ouchi H, Sato Y, Tsuneyama K, Kage M (2017). Acute presentation of autoimmune hepatitis: a multicentre study with detailed histological evaluation in a large cohort of patients. J Clin Pathol.

[102] Fujiwara K, Fukuda Y, Yokosuka O (2008). Precise histological evaluation of liver biopsy specimen is indispensable for diagnosis and treatment of acute-onset autoimmune hepatitis. J Gastroenterol.

[103] Fujiwara K, Nakano M, Yasui S, Okitsu K, Yonemitsu Y, Yokosuka O (2011). Advanced histology and impaired liver regeneration are associated with disease severity in acute-onset autoimmune hepatitis. Histopathology.

[104] Burgart LJ, Batts KP, Ludwig J, Nikias GA, Czaja AJ (1995). Recent-onset autoimmune hepatitis. Biopsy findings and clinical correlations. Am J Surg Pathol.

[105] Takahashi A, Arinaga-Hino T, Ohira H, Torimura T, Zeniya M, Abe M et al. Autoimmune hepatitis in Japan: trends in a nationwide survey. J Gastroenterol. 2017;52(5):631–640. 10.1007/s00535-016-1267-0 10.1007/s00535-016-1267-0 27722997

[106] Desmet VJ, Gerber M, Hoofnagle JH, Manns M, Scheuer PJ (1994). Classification of chronic hepatitis: diagnosis, grading and staging. Hepatology.

[107] de Boer YS, van Nieuwkerk CMJ, Witte BI, Mulder CJJ, Bouma G, Bloemena E (2015). Assessment of the histopathological key features in autoimmune hepatitis. Histopathology.

[108] K Washington, MP Manns (2018), Autoimmune Hepatitis. Macsween's Pathology of the Liver. AD Burt, LD Ferrell, SG Hübscher (Ed) 7th ed: Elsevier 491-514

[109] Gonzalez RS, Washington K (2018). Primary biliary cholangitis and autoimmune hepatitis. Surg Pathol Clin.

[110] Lefkowitch JH, Schiff ER, Davis GL, Perrillo RP, Lindsay K, Bodenheimer HC (1993). Pathological diagnosis of chronic hepatitis C: a multicenter comparative study with chronic hepatitis B. The hepatitis interventional therapy group. Gastroenterology.

[111] Bach N, Thung SN, Schaffner F (1992). The histological features of chronic hepatitis C and autoimmune chronic hepatitis: a comparative analysis. Hepatology.

[112] Suzuki A, Brunt EM, Kleiner DE, Miquel R, Smyrk TC, Andrade RJ (2011). The use of liver biopsy evaluation in discrimination of idiopathic autoimmune hepatitis versus drug-induced liver injury. Hepatology.

[113] Hisamochi A, Kage M, Ide T, Arinaga-Hino T, Amano K, Kuwahara R (2016). An analysis of drug-induced liver injury, which showed histological findings similar to autoimmune hepatitis. J Gastroenterol.

[114] De Martin E, Michot J-M, Papouin B, Champiat S, Mateus C, Lambotte O (2018). Characterization of liver injury induced by cancer immunotherapy using immune checkpoint inhibitors. J Hepatol.

[115] Kleiner DE, Chalasani NP, Lee WM, Fontana RJ, Bonkovsky HL, Watkins PB (2014). Hepatic histological findings in suspected drug-induced liver injury: systematic evaluation and clinical associations. Hepatology.

[116] Foureau DM, Walling TL, Maddukuri V, Anderson W, Culbreath K, Kleiner DE (2015). Comparative analysis of portal hepatic infiltrating leucocytes in acute drug-induced liver injury, idiopathic autoimmune and viral hepatitis. Clin Exp Immunol.

[117] Mietkiewski JM, Scheuer PJ (1985). Immunoglobulin-containing plasma cells in acute hepatitis. Liver.

[118] de Boer YS, Kosinski AS, Urban TJ, Zhao Z, Long N, Chalasani N (2017). Features of autoimmune hepatitis in patients with drug-induced liver injury. Clin Gastroenterol Hepatol.

[119] Balitzer D, Shafizadeh N, Peters MG, Ferrell LD, Alshak N, Kakar S (2017). Autoimmune hepatitis: review of histologic features included in the simplified criteria proposed by the international autoimmune hepatitis group and proposal for new histologic criteria. Mod Pathol..

[120] Tiniakos DG, Brain JG, Bury YA (2015). Role of histopathology in autoimmune hepatitis. Dig Dis.

[121] Miao Q, Bian Z, Tang R, Zhang H, Wang Q, Huang S (2015). Emperipolesis mediated by CD8 T cells is a characteristic histopathologic feature of autoimmune hepatitis. Clin Rev Allergy Immunol.

[122] Himoto T, Kadota K, Fujita K, Nomura T, Morishita A, Yoneyama H (2017). The pathological appearance of hyaline droplets in Kupffer cells is not specific to patients with autoimmune hepatitis. Int J Clin Exp Pathol.

[123] Lotowska JM, Sobaniec-Lotowska ME, Daniluk U, Lebensztejn DM (2017). Glassy droplet inclusions within the cytoplasm of Kupffer cells: a novel ultrastructural feature for the diagnosis of pediatric autoimmune hepatitis. Dig Liver Dis.

[124] Gurung A, Assis DN, McCarty TR, Mitchell KA, Boyer JL, Jain D (2018). Histologic features of autoimmune hepatitis: a critical appraisal. Hum Pathol.

[125] Aizawa Y, Abe H, Sugita T, Seki N, Chuganji Y, Furumoto Y (2016). Centrilobular zonal necrosis as a hallmark of a distinctive subtype of autoimmune hepatitis. Eur J Gastroenterol Hepatol.

[126] Kessler WR, Cummings OW, Eckert G, Chalasani N, Lumeng L, Kwo PY (2004). Fulminant hepatic failure as the initial presentation of acute autoimmune hepatitis. Clin Gastroenterol Hepatol.

[127] Abe K, Kanno Y, Okai K, Katsushima F, Monoe K, Saito H (2012). Centrilobular necrosis in acute presentation of Japanese patients with type 1 autoimmune hepatitis. World J Hepatol.

[128] Fujiwara K, Yasui S, Tawada A, Fukuda Y, Nakano M, Yokosuka O (2011). Diagnostic value and utility of the simplified international autoimmune hepatitis group criteria in acute-onset autoimmune hepatitis. Liver Int.

[129] Hofer H, Oesterreicher C, Wrba F, Ferenci P, Penner E (2006). Centrilobular necrosis in autoimmune hepatitis: a histological feature associated with acute clinical presentation. J Clin Pathol.

[130] Miyake Y, Iwasaki Y, Terada R, Onishi T, Okamoto R, Takaguchi K (2007). Clinical features of Japanese type 1 autoimmune hepatitis patients with zone III necrosis. Hepatol Res.

[131] Miao Q, Bian Z, Tang R, Zhang H, Wang Q, Huang S (2015). Emperipolesis mediated by CD8 T cells is a characteristic histopathologic feature of autoimmune hepatitis. Clin Rev Allergy Immunol.

[132] Te HS, Koukoulis G, Ganger DR (1997). Autoimmune hepatitis: a histological variant associated with prominent centrilobular necrosis. Gut.

[133] Pratt DS, Fawaz KA, Rabson A, Dellelis R, Kaplan MM (1997). A novel histological lesion in glucocorticoid-responsive chronic hepatitis. Gastroenterology.

[134] Ranvir S, Nair S, Farr G, Mason A, Perrillo R (2002). Acute autoimmune hepatitis presenting with centrizonal liver disease: case report and review of the literature. Am J Gastroenterol.

[135] Misdraji J, Thiim M, Graeme-Cook FM (2004). Autoimmune hepatitis with centrilobular necrosis. Am J Surg Pathol.

[136] Zen Y, Notsumata K, Tanaka N, Nakanuma Y (2007). Hepatic centrilobular zonal necrosis with positive antinuclear antibody: a unique subtype or early disease of autoimmune hepatitis?. Hum Pathol.

[137] Abe M, Onji M, Kawai-Ninomiya K, Michitaka K, Matsuura B, Hiasa Y (2007). Clinicopathologic features of the severe form of acute type 1 autoimmune hepatitis. Clin Gastroenterol Hepatol.

[138] Yoshizawa K, Joshita S, Matsumoto A, Umemura T, Tanaka E, Morita S (2016). Incidence and prevalence of autoimmune hepatitis in the Ueda area, Japan. Hepatol Res.

[139] Fujiwara K, Fukuda Y, Seza K, Saito M, Yasui S, Nakano M (2016). High level of persistent liver injury is one of clinical characteristics in treatment-naïve acute onset autoimmune hepatitis: experience in a community hospital. J Hepatobiliary Pancreat Sci.

[140] Fujiwara K, Yasui S, Yokosuka O (2013). Autoimmune acute liver failure: an emerging etiology for intractable acute liver failure. Hepatol Int.

[141] Okano N, Yamamoto K, Sakaguchi K, Miyake Y, Shimada N, Hakoda T (2003). Clinicopathological features of acute-onset autoimmune hepatitis. Hepatol Res.

[142] Fujiwara K, Yasui S, Tawada A, Okitsu K, Yonemitsu Y, Chiba T (2011). Autoimmune fulminant liver failure in adults: experience in a Japanese center. Hepatol Res.

[143] Fujiwara K, Yasui S, Yonemitsu Y, Arai M, Kanda T, Fukuda Y (2016). Analysis of infectious complications and timing for emergency liver transplantation in autoimmune acute liver failure. J Hepatobiliary Pancreat Sci.

[144] Nikias GA, Batts KP, Czaja AJ (1994). The nature and prognostic implications of autoimmune hepatitis with an acute presentation. J Hepatol.

[145] Yasui S, Fujiwara K, Yonemitsu Y, Oda S, Nakano M, Yokosuka O (2011). Clinicopathological features of severe and fulminant forms of autoimmune hepatitis. J Gastroenterol.

[146] Ferrari R, Pappas G, Agostinelli D, Muratori P, Muratori L, Lenzi M (2004). Type 1 autoimmune hepatitis: patterns of clinical presentation and differential diagnosis of the ‘acute’ type. QJM.

[147] van Gerven NM, Verwer BJ, Witte BI, van Erpecum KJ, van Buuren HR, Maijers I (2014). Epidemiology and clinical characteristics of autoimmune hepatitis in the Netherlands. Scand J Gastroenterol.

[148] Than NN, Ching DK, Hodson J, McDowell P, Mann J, Gupta R (2016). Difference in clinical presentation, immunology profile and treatment response of type 1 autoimmune hepatitis between United Kingdom and Singapore patients. Hepatol Int.

[149] Takahashi A, Arinaga-Hino T, Ohira H, Torimura T, Zeniya M, Abe M (2017). Autoimmune hepatitis in Japan: trends in a nationwide survey. J Gastroenterol.

[150] Burt AD, Lackner C, Tiniakos DG (2015). Diagnosis and assessment of NAFLD: definitions and histopathological classification. Semin Liver Dis.

[151] Wang Z, Sheng L, Yang Y, Yang F, Xiao X, Hua J (2017). The Management of autoimmune hepatitis patients with decompensated cirrhosis: real-world experience and a comprehensive review. Clin Rev Allergy Immunol.

[152] Wang QX, Jiang WJ, Miao Q, Xiao X, Zhang HY, Huang SS (2013). Clinical and histological features of autoantibody-negative autoimmune hepatitis in Chinese patients: a single center experience. J Dig Dis.

[153] Baeres M, Herkel J, Czaja AJ, Wies I, Kanzler S, Cancado EL (2002). Establishment of standardised SLA/LP immunoassays: specificity for autoimmune hepatitis, worldwide occurrence, and clinical characteristics. Gut.

[154] Efe C, Ozaslan E, Wahlin S, Purnak T, Muratori L, Quarneti C (2013). Antibodies to soluble liver antigen in patients with various liver diseases: a multicentre study. Liver Int.

[155] Umemura T, Joshita S, Saito H, Yoshizawa K, Norman GL, Tanaka E (2019). KIR/HLA genotypes confer susceptibility and progression in patients with autoimmune hepatitis. JHEP Rep.

[156] Yilmaz B, Unlu O, Evcen R, Ugurluoglu C (2016). Acute onset seronegative autoimmune hepatitis: are simplified diagnostic criteria sufficient?. Eur J Gastroenterol Hepatol.

[157] Czaja AJ (1999). Behavior and significance of autoantibodies in type 1 autoimmune hepatitis. J Hepatol.

[158] Poynard T, Ngo Y, Perazzo H, Munteanu M, Lebray P, Moussalli J (2011). Prognostic value of liver fibrosis biomarkers: a meta-analysis. Gastroenterol Hepatol (N Y).

[159] Wai CT, Greenson JK, Fontana RJ, Kalbfleisch JD, Marrero JA, Conjeevaram HS (2003). A simple noninvasive index can predict both significant fibrosis and cirrhosis in patients with chronic hepatitis C. Hepatology.

[160] Shah AG, Lydecker A, Murray K, Tetri BN, Contos MJ, Sanyal AJ (2009). Comparison of noninvasive markers of fibrosis in patients with nonalcoholic fatty liver disease. Clin Gastroenterol Hepatol.

[161] MS Gungoren, C Efe, T Kav, F Akbiyik (2018) Diagnostic accuracy of enhanced liver fibrosis (ELF) test for significant fibrosis in patients with autoimmune hepatitis. J Lab Precis Med **3**(3)

[162] Efe C, Cengiz M, Kahramanoğlu-Aksoy E, Yilmaz B, Özşeker B, Beyazt Y (2015). Angiotensin-converting enzyme for noninvasive assessment of liver fibrosis in autoimmune hepatitis. Eur J Gastroenterol Hepatol.

[163] Liu L, Cao J, Zhong Z, Guo Z, Jiang Y, Bai Y (2019). Noninvasive indicators predict advanced liver fibrosis in autoimmune hepatitis patients. J Clin Lab Anal.

[164] Yuan X, Duan SZ, Cao J, Gao N, Xu J, Zhang L (2019). Noninvasive inflammatory markers for assessing liver fibrosis stage in autoimmune hepatitis patients. Eur J Gastroenterol Hepatol.

[165] Zeng T, Yu J, Tan L, Wu Y, Tian Y, Wu Q (2018). Noninvasive indices for monitoring disease course in Chinese patients with autoimmune hepatitis. Clin Chim Acta.

[166] Wang QX, Shen L, Qiu DK, Bao H, Chen XY, Zeng MD (2011). Validation of transient elastography (Fibroscan) in assessment of hepatic fibrosis in autoimmune hepatitis. Zhonghua Gan Zang Bing Za Zhi.

[167] Obara N, Ueno Y, Fukushima K, Nakagome Y, Kakazu E, Kimura O (2008). Transient elastography for measurement of liver stiffness measurement can detect early significant hepatic fibrosis in Japanese patients with viral and nonviral liver diseases. J Gastroenterol.

[168] Efe C, Gungoren MS, Ozaslan E, Akbiyik F, Kav T (2015). Acoustic radiation force impulse (ARFI) for fibrosis staging in patients with autoimmune hepatitis. Hepatogastroenterology.

[169] Goertz RS, GaBmann L, Strobel D, Wildner D, Schellhaas B, Neurath MF (2019). Acoustic radiation force impulse (ARFI) elastography in autoimmune and cholestatic liver diseases. Ann Hepatol.

[170] Wang J, Malik N, Yin M, Smyrk TC, Czaja AJ, Ehman RL (2017). Magnetic resonance elastography is accurate in detecting advanced fibrosis in autoimmune hepatitis. World J Gastroenterol.

[171] Czaja AJ (2008). Performance parameters of the diagnostic scoring systems for autoimmune hepatitis. Hepatology.

[172] Johnson PJ, McFarlane IG (1993). Meeting report: international autoimmune hepatitis group. Hepatology.

[173] Miyake Y, Iwasaki Y, Kobashi H, Yasunaka T, Ikeda F, Takaki A (2010). Clinical features of autoimmune hepatitis diagnosed based on simplified criteria of the international autoimmune hepatitis group. Dig Liver Dis.

[174] Hennes EM, Zeniya M, Czaja AJ, Pares A, Dalekos GN, Krawitt EL (2008). Simplified criteria for the diagnosis of autoimmune hepatitis. Hepatology.

[175] Yeoman AD, Westbrook RH, Al-Chalabi T, Carey I, Heaton ND, Portmann BC (2009). Diagnostic value and utility of the simplified international autoimmune hepatitis group (IAIHG) criteria in acute and chronic liver disease. Hepatology.

[176] Qiu D, Wang Q, Wang H, Xie Q, Zang G, Jiang H (2011). Validation of the simplified criteria for diagnosis of autoimmune hepatitis in Chinese patients. J Hepatol.

[177] Kim BH, Kim YJ, Jeong SH, Tak WY, Ahn SH, Lee YJ (2013). Clinical features of autoimmune hepatitis and comparison of two diagnostic criteria in Korea: a nationwide, multicenter study. J Gastroenterol Hepatol.

[178] Chazouilleres O, Wendum D, Serfaty L, Montembault S, Rosmorduc O, Poupon R (1998). Primary biliary cirrhosis-autoimmune hepatitis overlap syndrome: clinical features and response to therapy. Hepatology.

[179] Wang Q, Selmi C, Zhou X, Qiu D, Li Z, Miao Q (2013). Epigenetic considerations and the clinical reevaluation of the overlap syndrome between primary biliary cirrhosis and autoimmune hepatitis. J Autoimmun.

[180] Chapman R, Fevery J, Kalloo A, Nagorney DM, Boberg KM, Shneider B (2010). Diagnosis and management of primary sclerosing cholangitis. Hepatology.

[181] Kaya M, Angulo P, Lindor KD (2000). Overlap of autoimmune hepatitis and primary sclerosing cholangitis: an evaluation of a modified scoring system. J Hepatol.

[182] Lian M, Li B, Xiao X, Yang Y, Jiang P, Yan L (2017). Comparative clinical characteristics and natural history of three variants of sclerosing cholangitis: IgG4-related SC, PSC/AIH and PSC alone. Autoimmun Rev.

[183] deLemos AS, Foureau DM, Jacobs C, Ahrens W, Russo MW, Bonkovsky HL (2014). Drug-induced liver injury with autoimmune features. Semin Liver Dis.

[184] Tsuneyama K, Baba H, Kikuchi K, Nishida T, Nomoto K, Hayashi S (2013). Autoimmune features in metabolic liver disease: a single-center experience and review of the literature. Clin Rev Allergy Immunol.

[185] Takahashi A, Arinaga-Hino T, Ohira H, Abe K, Torimura T, Zeniya M (2018). Non-alcoholic fatty liver disease in patients with autoimmune hepatitis. JGH Open.

[186] Taubert R, Diestelhorst J, Junge N, Kirstein MM, Pischke S, Vogel A (2018). Increased seroprevalence of HAV and parvovirus B19 in children and of HEV in adults at diagnosis of autoimmune hepatitis. Sci Rep.

[187] Pischke S, Gisa A, Suneetha PV, Wiegand SB, Taubert R, Schlue J (2014). Increased HEV seroprevalence in patients with autoimmune hepatitis. PLoS ONE.

[188] Sener AG, Aydin N, Ceylan C, Kirdar S (2018). Investigation of antinuclear antibodies in chronic hepatitis B patients. Mikrobiyol Bul.

[189] Gilman AJ, Le AK, Zhao C, Hoang J, Yasukawa LA, Weber SC (2018). Autoantibodies in chronic hepatitis C virus infection: impact on clinical outcomes and extrahepatic manifestations. BMJ Open Gastroenterol.

[190] Sui M, Wu R, Hu X, Zhang H, Jiang J, Yang Y (2014). Low prevalence of hepatitis B virus infection in patients with autoimmune diseases in a Chinese patient population. J Viral Hepat.

[191] Shantha S, Thyagarajan SP, Premavathy RK, Sukumar RG, Mohan KV, Palanisamy KR (2002). Correlation of autoimmune reactivity with hepatitis B and C virus (HBV and HCV) infection in histologically proven chronic liver diseases. Indian J Med Microbiol.

[192] Efe C, Wahlin S, Ozaslan E, Purnak T, Muratori L, Quarneti C (2013). Diagnostic difficulties, therapeutic strategies, and performance of scoring systems in patients with autoimmune hepatitis and concurrent hepatitis B/C. Scand J Gastroenterol.

[193] Yener S, Akarsu M, Karacanci C, Sengul B, Topalak O, Biberoglu K (2004). Wilson’s disease with coexisting autoimmune hepatitis. J Gastroenterol Hepatol.

[194] Acharya GK, Liao HI, Frunza-Stefan S, Patel R, Khaing M (2017). Autoimmune hepatitis: diagnostic dilemma when it is disguised as iron overload syndrome. J Clin Exp Hepatol.

[195] Hohler T, Leininger S, Kohler HH, Schirmacher P, Galle PR (2000). Heterozygosity for the hemochromatosis gene in liver diseases–prevalence and effects on liver histology. Liver.

[196] Efe C, Ozaslan E, Heurgue-Berlot A, Kav T, Masi C, Purnak T (2014). Sequential presentation of primary biliary cirrhosis and autoimmune hepatitis. Eur J Gastroenterol Hepatol.

[197] Boberg KM, Fausa O, Haaland T, Holter E, Mellbye OJ, Spurkland A (1996). Features of autoimmune hepatitis in primary sclerosing cholangitis: an evaluation of 114 primary sclerosing cholangitis patients according to a scoring system for the diagnosis of autoimmune hepatitis. Hepatology.

[198] Cook GC, Mulligan R, Sherlock S (1971). Controlled prospective trial of corticosteroid therapy in active chronic hepatitis. Q J Med.

[199] Murray-Lyon I, Stern R, Williams R (1973). Controlled trial of prednisone and azathioprine in active chronic hepatitis. Lancet.

[200] Soloway R, Summerskill W, Baggenstoss A, Geall M, Gitnick G, Elveback I (1972). Clinical, biochemical, and histological remission of severe chronic active liver disease: a controlled study of treatments and early prognosis. Gastroenterology.

[201] Summerskill W, Korman M, Ammon H, Baggenstoss A (1975). Prednisone for chronic active liver disease: dose titration, standard dose, and combination with azathioprine compared. Gut.

[202] Tage-Jensen U, Schlichting P, Aldershvile J, Andersen P, Dietrichson O, Hardt F (1982). Azathioprine versus prednisone in non-alcoholic chronic liver disease (CLD). Relation to a serological classification. Liver.

[203] Lamers MM, van Oijen MG, Pronk M, Drenth JP (2010). Treatment options for autoimmune hepatitis: a systematic review of randomized controlled trials. J Hepatol.

[204] Baggenstoss AH, Soloway RD, Summerskill WH, Elveback LR, Schoenfield LJ (1972). Chronic active liver disease. The range of histologic lesions, their response to treatment, and evolution. Hum Pathol.

[205] Cooksley WG, Bradbear RA, Robinson W, Harrison M, Halliday JW, Powell LW (1986). The prognosis of chronic active hepatitis without cirrhosis in relation to bridging necrosis. Hepatology.

[206] Yoshizawa K, Matsumoto A, Ichijo T, Umemura T, Joshita S, Komatsu M (2012). Long-term outcome of Japanese patients with type 1 autoimmune hepatitis. Hepatology.

[207] Amarapurkar D, Dharod M, Amarapurkar A (2015). Autoimmune hepatitis in India: single tertiary referral centre experience. Trop Gastroenterol.

[208] Muratori L, Muratori P, Lanzoni G, Ferri S, Lenzi M (2010). Application of the 2010 American association for the study of liver diseases criteria of remission to a cohort of Italian patients with autoimmune hepatitis. Hepatology.

[209] Luth S, Herkel J, Kanzler S, Frenzel C, Galle PR, Dienes HP (2008). Serologic markers compared with liver biopsy for monitoring disease activity in autoimmune hepatitis. J Clin Gastroenterol.

[210] Pape S, Gevers TJG, Vrolijk JM, van Hoek B, Bouma G, van Nieuwkerk CMJ (2020). Rapid response to treatment of autoimmune hepatitis associated with remission at 6 and 12 months. Clin Gastroenterol Hepatol.

[211] Dyson JK, De Martin E, Dalekos GN, Drenth JPH, Herkel J, Hubscher SG (2019). Review article: unanswered clinical and research questions in autoimmune hepatitis-conclusions of the international autoimmune hepatitis group research workshop. Aliment Pharmacol Ther.

[212] Schramm C, Wahl I, Weiler-Normann C, Voigt K, Wiegard C, Glaubke C (2014). Health-related quality of life, depression, and anxiety in patients with autoimmune hepatitis. J Hepatol.

[213] Sockalingam S, Blank D, Abdelhamid N, Abbey SE, Hirschfield GM (2012). Identifying opportunities to improve management of autoimmune hepatitis: evaluation of drug adherence and psychosocial factors. J Hepatol.

[214] Czaja AJ (2009). Features and consequences of untreated type 1 autoimmune hepatitis. Liver Int.

[215] Czaja AJ, Carpenter HA (2004). Decreased fibrosis during corticosteroid therapy of autoimmune hepatitis. J Hepatol.

[216] Czaja AJ (2014). Hepatic inflammation and progressive liver fibrosis in chronic liver disease. World J Gastroenterol.

[217] Czaja AJ (2014). Review article: the prevention and reversal of hepatic fibrosis in autoimmune hepatitis. Aliment Pharmacol Ther.

[218] Beretta-Piccoli BT, Mieli-Vergani G, Vergani D (2017). Autoimmune hepatitis: Standard treatment and systematic review of alternative treatments. World J Gastroenterol.

[219] Orlando R, Lirussi F, Okolicsanyi L (1990). Laparoscopy and liver biopsy: further evidence that the two procedures improve the diagnosis of liver cirrhosis. A retrospective study of 1003 consecutive examinations. J Clin Gastroenterol.

[220] Kirk AP, Jain S, Pocock S, Thomas HC, Sherlock S (1980). Late results of the royal free hospital prospective controlled trial of prednisolone therapy in hepatitis B surface antigen negative chronic active hepatitis. Gut.

[221] Yeoman AD, Westbrook RH, Zen Y, Bernal W, Al-Chalabi T, Wendon JA (2014). Prognosis of acute severe autoimmune hepatitis (AS-AIH): the role of corticosteroids in modifying outcome. J Hepatol.

[222] Czaja AJ (2009). Rapidity of treatment response and outcome in type 1 autoimmune hepatitis. J Hepatol.

[223] Purnak T, Efe C, Kav T, Wahlin S, Ozaslan E (2017). Treatment response and outcome with two different prednisolone regimens in autoimmune hepatitis. Dig Dis Sci.

[224] Zhang C, Wu SS, Dong XQ, Wu Z, Zhao H, Wang GQ (2019). The efficacy and safety of different doses of glucocorticoid for autoimmune hepatitis: a systematic review and meta-analysis. Medicine (Baltimore).

[225] Pape S, Gevers TJG, Belias M, Mustafajev IF, Vrolijk JM, van Hoek B (2019). Predniso(lo)ne dosage and chance of remission in patients with autoimmune hepatitis. Clin Gastroenterol Hepatol.

[226] Pape S, Gevers TJG, Vrolijk JM, van Hoek B, Bouma G, van Nieuwkerk CMJ (2020). High discontinuation rate of azathioprine in autoimmune hepatitis, independent of time of treatment initiation. Liver Int.

[227] Newman WG, Payne K, Tricker K, Roberts SA, Fargher E, Pushpakom S (2011). A pragmatic randomized controlled trial of thiopurine methyltransferase genotyping prior to azathioprine treatment: the TARGET study. Pharmacogenomics.

[228] American Gastroenterological Association (2003). American Gastroenterological Association medical position statement: osteoporosis in hepatic disorders. Gastroenterology.

[229] Wong LL, Fisher HF, Stocken DD, Rice S, Khanna A, Heneghan MA (2018). The impact of autoimmune hepatitis and its treatment on health utility. Hepatology.

[230] van den Brand FF, van der Veen KS, Lissenberg-Witte BI, de Boer YS, van Hoek B, Drenth JPH (2019). Adverse events related to low dose corticosteroids in autoimmune hepatitis. Aliment Pharmacol Ther.

[231] Weinshilboum RM, Sladek SL (1980). Mercaptopurine pharmacogenetics: monogenic inheritance of erythrocyte thiopurine methyltransferase activity. Am J Hum Genet.

[232] Davavala SK, Desai DC, Abraham P, Ashavaid T, Joshi A, Gupta T (2014). Prevalence of TPMT polymorphism in Indian patients requiring immunomodulator therapy and its clinical significance. Indian J Gastroenterol.

[233] Kumagai K, Hiyama K, Ishioka S, Sato H, Yamanishi Y, McLeod HL (2001). Allelotype frequency of the thiopurine methyltransferase (TPMT) gene in Japanese. Pharmacogenetics.

[234] Cao Q, Zhu Q, Shang Y, Gao M, Si J (2009). Thiopurine methyltransferase gene polymorphisms in Chinese patients with inflammatory bowel disease. Digestion.

[235] Takatsu N, Matsui T, Murakami Y, Ishihara H, Hisabe T, Nagahama T (2009). Adverse reactions to azathioprine cannot be predicted by thiopurine S-methyltransferase genotype in Japanese patients with inflammatory bowel disease. J Gastroenterol Hepatol.

[236] Yang SK, Hong M, Baek J, Choi H, Zhao W, Jung Y (2014). A common missense variant in NUDT15 confers susceptibility to thiopurine-induced leukopenia. Nat Genet.

[237] Chao K, Wang X, Cao Q, Qian J, Wu K, Zhu X (2017). Combined detection of NUDT15 variants could highly predict thiopurine-induced leukopenia in Chinese Patients with inflammatory bowel disease: a multicenter analysis. Inflamm Bowel Dis.

[238] Kim HT, Choi R, Won HH, Choe YH, Kang B, Lee K (2017). NUDT15 genotype distributions in the Korean population. Pharmacogenet Genomics.

[239] Kuriyama S, Yaegashi N, Nagami F, Arai T, Kawaguchi Y, Osumi N (2016). The Tohoku medical megabank project: design and mission. J Epidemiol.

[240] Langley PG, Underhill J, Tredger JM, Norris S, McFarlane IG (2002). Thiopurine methyltransferase phenotype and genotype in relation to azathioprine therapy in autoimmune hepatitis. J Hepatol.

[241] Kakuta Y, Naito T, Onodera M, Kuroha M, Kimura T, Shiga H (2016). NUDT15 R139C causes thiopurine-induced early severe hair loss and leukopenia in Japanese patients with IBD. Pharmacogenomics J.

[242] Karakoyun M, Ecevit CO, Kilicoglu E, Aydogdu S, Yagci RV, Ozgenc F (2016). Autoimmune hepatitis and long-term disease course in children in Turkey, a single-center experience. Eur J Gastroenterol Hepatol.

[243] Heneghan MA, Allan ML, Bornstein JD, Muir AJ, Tendler DA (2006). Utility of thiopurine methyltransferase genotyping and phenotyping, and measurement of azathioprine metabolites in the management of patients with autoimmune hepatitis. J Hepatol.

[244] Johnson PJ, McFarlane IG, Williams R (1995). Azathioprine for long-term maintenance of remission in autoimmune hepatitis. N Engl J Med.

[245] Lohse AW, Mieli-Vergani G (2011). Autoimmune hepatitis. J Hepatol.

[246] Pratt DS, Flavin DP, Kaplan MM (1996). The successful treatment of autoimmune hepatitis with 6-mercaptopurine after failure with azathioprine. Gastroenterology.

[247] Akbari M, Shah S, Velayos FS, Mahadevan U, Cheifetz AS (2013). Systematic review and meta-analysis on the effects of thiopurines on birth outcomes from female and male patients with inflammatory bowel disease. Inflamm Bowel Dis.

[248] Mack CL, Adams D, Assis DN, Kerkar N, Manns MP, Mayo MJ (2020). Diagnosis and management of autoimmune hepatitis in adults and children: 2019 practice guidance and guidelines from the American association for the study of liver diseases. Hepatology.

[249] Angelberger S, Reinisch W, Messerschmidt A, Miehsler W, Novacek G, Vogelsang H (2011). Long-term follow-up of babies exposed to azathioprine in utero and via breastfeeding. J Crohns Colitis.

[250] Miyake Y, Iwasaki Y, Terada R, Okamaoto R, Ikeda H, Makino Y (2006). Persistent elevation of serum alanine aminotransferase levels leads to poor survival and hepatocellular carcinoma development in type 1 autoimmune hepatitis. Aliment Pharmacol Ther.

[251] Hoeroldt B, McFarlane E, Dube A, Basumani P, Karajeh M, Campbell MJ (2011). Long-term outcomes of patients with autoimmune hepatitis managed at a nontransplant center. Gastroenterology.

[252] Hegarty JE, Aria KTN, Portmann B, Eddleston AL, Williams R (1983). Relapse following treatment withdrawal in patients with autoimmune chronic active hepatitis. Hepatology.

[253] van Gerven NM, Verwer BJ, Witte BI, van Hoek B, Coenraad MJ, van Erpecum KJ (2013). Relapse is almost universal after withdrawal of immunosuppressive medication in patients with autoimmune hepatitis in remission. J Hepatol.

[254] Czaja AJ (2010). Late relapse of type 1 autoimmune hepatitis after corticosteroid withdrawal. Dig Dis Sci.

[255] Hartl J, Ehlken H, Weiler-Normann C, Sebode M, Kreuels B, Pannicke N (2015). Patient selection based on treatment duration and liver biochemistry increases success rates after treatment withdrawal in autoimmune hepatitis. J Hepatol.

[256] Montano-Loza AJ, Carpenter HA, Czaja AJ (2007). Improving the end point of corticosteroid therapy in type 1 autoimmune hepatitis to reduce the frequency of relapse. Am J Gastroenterol.

[257] Efe C, Simşek C, Batıbay E, Calışkan AR, Wahlin S (2020). Feasibility of telehealth in the management of autoimmune hepatitis before and during the COVID-19 pandemic. Expert Rev Gastroenterol Hepatol.

[258] Schalm SW, Ammon HV, Summerskill WH (1976). Failure of customary treatment in chronic active liver disease: causes and management. Ann Clin Res.

[259] Hindorf U, Jahed K, Bergquist A, Verbaan H, Prytz H, Wallerstedt S (2010). Characterisation and utility of thiopurine methyltransferase and thiopurine metabolite measurements in autoimmune hepatitis. J Hepatol.

[260] Czaja AJ, Wolf AM, Baggenstoss AH (1981). Laboratory assessment of severe chronic active liver disease during and after corticosteroid therapy: correlation of serum transaminase and gamma globulin levels with histologic features. Gastroenterology.

[261] Czaja AJ (2008). Safety issues in the management of autoimmune hepatitis. Expert Opin Drug Saf.

[262] Hennes EM, Oo YH, Schramm C, Denzer U, Buggisch P, Wiegard C (2008). Mycophenolate mofetil as second line therapy in autoimmune hepatitis?. Am J Gastroenterol.

[263] Vergani D, Mieli-Vergani G (2011). Pharmacological management of autoimmune hepatitis. Expert Opin Pharmacother.

[264] Czaja AJ, Menon KV, Carpenter HA (2002). Sustained remission after corticosteroid therapy for type 1 autoimmune hepatitis: a retrospective analysis. Hepatology.

[265] Duclos-Vallee JC, Ichai P, Samuel D (2011). Autoimmune acute liver failure. Hepatology.

[266] Moenne-Loccoz R, Severac F, Baumert TF, Habersetzer F (2016). Usefulness of corticosteroids as first-line therapy in patients with acute severe autoimmune hepatitis. J Hepatol.

[267] Zhu B, You SL, Wan ZH, Liu HL, Rong YH, Zang H (2014). Clinical characteristics and corticosteroid therapy in patients with autoimmune-hepatitis-induced liver failure. World J Gastroenterol.

[268] Ichai P, Duclos-Vallee JC, Guettier C, Hamida SB, Antonini T, Delvart V (2007). Usefulness of corticosteroids for the treatment of severe and fulminant forms of autoimmune hepatitis. Liver Transpl.

[269] Czaja AJ (2007). Corticosteroids or not in severe acute or fulminant autoimmune hepatitis: therapeutic brinksmanship and the point beyond salvation. Liver Transpl.

[270] Miyake Y, Iwasaki Y, Terada R, Onishi T, Okamoto R, Sakai N (2006). Clinical characteristics of fulminant-type autoimmune hepatitis: an analysis of eleven cases. Aliment Pharmacol Ther.

[271] Yeoman AD, Westbrook RH, Zen Y, Maninchedda P, Portmann BC, Devlin J (2011). Early predictors of corticosteroid treatment failure in icteric presentations of autoimmune hepatitis. Hepatology.

[272] Li YN, Ma H, Zhou L, Zhang J, Guo LP, Li SQ (2016). Autoimmune hepatitis-related cirrhosis: clinical features and effectiveness of immunosuppressive treatment in Chinese patients. Chin Med J (Engl).

[273] Ng TL et al. Immunosuppresive therapy in autoimmune hepatitis in Hong Kong Chinese: 10-year follow-up of a cohort. Hepatol Int 2009;(Suppl):86–220. 10.1007/s12072-009-9123-4

[274] Wang QX, Yan L, Ma X (2018). Autoimmune hepatitis in the Asia–Pacific area. J Clin Transl Hepatol.

[275] Soares JC, Borgonovo A, Maggi DC, Pasinato AP, Ramos FG, Dantas-Correa EB (2016). Liver dysfunction and fibrosis as predictors of biochemical response to autoimmune hepatitis treatment. Minerva Gastroenterol Dietol.

[276] Efe C, Ozaslan E, Kav T, Purnak T, Shorbagi A, Ozkayar O (2012). Liver fibrosis may reduce the efficacy of budesonide in the treatment of autoimmune hepatitis and overlap syndrome. Autoimmun Rev.

[277] Tansel A, Katz LH, El-Serag HB, Thrift AP, Parepally M, Shakhatreh MH (2017). Incidence and determinants of hepatocellular carcinoma in autoimmune hepatitis: a systematic review and meta-analysis. Clin Gastroenterol Hepatol.

[278] Westbrook RH, Yeoman AD, Kriese S, Heneghan MA (2012). Outcomes of pregnancy in women with autoimmune hepatitis. J Autoimmun.

[279] Terrabuio DR, Abrantes-Lemos CP, Carrilho FJ, Cançado EL (2009). Follow-up of pregnant women with autoimmune hepatitis: the disease behavior along with maternal and fetal outcomes. J Clin Gastroenterol.

[280] CDC (2017) National birth defects prevention study (NBDPS). https://www.cdc.gov/ncbddd/birthdefects/nbdps.html. Accessed 26 Oct 2020

[281] Buchel E, Van Steenbergen W, Nevens F, Fevery J (2002). Improvement of autoimmune hepatitis during pregnancy followed by flare-up after delivery. Am J Gastroenterol.

[282] Gregorio GV, Portmann B, Karani J, Harrison P, Donaldson PT, Vergani D (2001). Autoimmune hepatitis/sclerosing cholangitis overlap syndrome in childhood: a 16-year prospective study. Hepatology.

[283] Gregorio GV, Portmann B, Reid F, Donaldson PT, Doherty DG, McCartney M (1997). Autoimmune hepatitis in childhood: a 20-year experience. Hepatology.

[284] Maggiore G, Veber F, Bernard O, Hadchouel M, Homberg JC, Alvarez F (1993). Autoimmune hepatitis associated with anti-actin antibodies in children and adolescents. J Pediatr Gastroenterol Nutr.

[285] Schramm C, Kanzler S, zum Buschenfelde KH, Galle PR, Lohse AW (2001). Autoimmune hepatitis in the elderly. Am J Gastroenterol.

[286] Rizvi S, Gawrieh S (2018). Autoimmune hepatitis in the elderly: diagnosis and pharmacologic management. Drugs Aging.

[287] De Lemos-Bonotto M, Valle-Tovo C, Costabeber AM, Mattos AA, Azeredo-da-Silva ALF (2018). A systematic review and meta-analysis of second-line immunosuppressants for autoimmune hepatitis treatment. Eur J Gastroenterol Hepatol.

[288] Zandieh I, Krygier D, Wong V, Howard J, Worobetz L, Minuk G (2008). The use of budesonide in the treatment of autoimmune hepatitis in Canada. Can J Gastroenterol.

[289] Hempfling W, Grunhage F, Dilger K, Reichel C, Beuers U, Sauerbruch T (2003). Pharmacokinetics and pharmacodynamic action of budesonide in early- and late-stage primary biliary cirrhosis. Hepatology.

[290] Csepregi A, Rocken C, Treiber G, Malfertheiner P (2006). Budesonide induces complete remission in autoimmune hepatitis. World J Gastroenterol.

[291] Manns MP, Woynarowski M, Kreisel W, Lurie Y, Rust C, Zuckerman E (2010). Budesonide induces remission more effectively than prednisone in a controlled trial of patients with autoimmune hepatitis. Gastroenterology.

[292] Czaja AJ (2009). Current and future treatments of autoimmune hepatitis. Expert Rev Gastroenterol Hepatol.

[293] Czaja AJ (2011). Promising pharmacological, molecular and cellular treatments of autoimmune hepatitis. Curr Pharm Des.

[294] De Maeyer F, Lapauw B, Hoorens A, Geerts A, Van Vlierberghe H, Verhelst X (2018). Secondary adrenal insufficiency after treatment with budesonide for autoimmune hepatitis. Case Rep Gastroenterol.

[295] Lu FB, Hu ED, Xu LM, Hu YB, Chen L, Wu JL (2018). Comparative efficacy and tolerability of treatments for adult autoimmune hepatitis: a systematic review and network meta-analysis. Exp Ther Med.

[296] Czaja AJ, Lindor KD (2000). Failure of budesonide in a pilot study of treatment-dependent autoimmune hepatitis. Gastroenterology.

[297] Zachou K, Gatselis NK, Arvaniti P, Gabeta S, Rigopoulou EI, Koukoulis GK (2016). A real-world study focused on the long-term efficacy of mycophenolate mofetil as first-line treatment of autoimmune hepatitis. Aliment Pharmacol Ther.

[298] Zolfino T, Heneghan MA, Norris S, Harrison PM, Portmann BC, McFarlane IG (2002). Characteristics of autoimmune hepatitis in patients who are not of European Caucasoid ethnic origin. Gut.

[299] Chatur N, Ramji A, Bain VG, Ma MM, Marotta PJ, Ghent CN (2005). Transplant immunosuppressive agents in non-transplant chronic autoimmune hepatitis: the Canadian association for the study of liver (CASL) experience with mycophenolate mofetil and tacrolimus. Liver Int.

[300] Jothimani D, Cramp ME, Cross TJ (2014). Role of mycophenolate mofetil for the treatment of autoimmune hepatitis-an observational study. J Clin Exp Hepatol.

[301] Zachou K, Gatselis N, Papadamou G, Rigopoulou EI, Dalekos GN (2011). Mycophenolate for the treatment of autoimmune hepatitis: prospective assessment of its efficacy and safety for induction and maintenance of remission in a large cohort of treatment-naive patients. J Hepatol.

[302] Giannakopoulos G, Verbaan H, Friis-Liby IL, Sangfelt P, Nyhlin N, Almer S (2019). Mycophenolate mofetil treatment in patients with autoimmune hepatitis failing standard therapy with prednisolone and azathioprine. Dig Liver Dis.

[303] Roberts SK, Lim R, Strasser S, Nicoll A, Gazzola A, Mitchell J (2018). Efficacy and safety of mycophenolate mofetil in patients with autoimmune hepatitis and suboptimal outcomes after standard therapy. Clin Gastroenterol Hepatol.

[304] Efe C, Hagstrom H, Ytting H, Bhanji RA, Muller NF, Wang Q (2017). Efficacy and safety of mycophenolate mofetil and tacrolimus as second-line therapy for patients with autoimmune hepatitis. Clin Gastroenterol Hepatol.

[305] Nasseri-Moghaddam S, Nikfam S, Karimian S, Khashayar P, Malekzadeh R (2013). Cyclosporine-A versus prednisolone for induction of remission in auto-immune hepatitis: interim analysis report of a randomized controlled trial. Middle East J Dig Dis.

[306] Hanouneh M, Ritchie MM, Ascha M, Ascha MS, Chedid A, Sanguankeo A (2019). A review of the utility of tacrolimus in the management of adults with autoimmune hepatitis. Scand J Gastroenterol.

[307] Than NN, Wiegard C, Weiler-Normann C, Fussel K, Mann J, Hodson J (2016). Long-term follow-up of patients with difficult to treat type 1 autoimmune hepatitis on tacrolimus therapy. Scand J Gastroenterol.

[308] Hubener S, Oo YH, Than NN, Hubener P, Weiler-Normann C, Lohse AW (2016). Efficacy of 6-mercaptopurine as second-line treatment for patients with autoimmune hepatitis and azathioprine intolerance. Clin Gastroenterol Hepatol.

[309] de Boer NK, van Nieuwkerk CM, Pages MNA, de Boer SY, Derijks LJ, Mulder CJ (2005). Promising treatment of autoimmune hepatitis with 6-thioguanine after adverse events on azathioprine. Eur J Gastroenterol Hepatol.

[310] Pavanello F, Zucca E, Ghielmini M (2017). Rituximab: 13 open questions after 20 years of clinical use. Cancer Treat Rev.

[311] Weiler-Normann C, Schramm C, Quaas A, Wiegard C, Glaubke C, Pannicke N (2013). Infliximab as a rescue treatment in difficult-to-treat autoimmune hepatitis. J Hepatol.

[312] Efe C (2013). Drug induced autoimmune hepatitis and TNF-α blocking agents: is there a real relationship?. Autoimmun Rev.

[313] Kerkar N, Dugan C, Rumbo C, Morotti RA, Gondolesi G, Shneider BL (2005). Rapamycin successfully treats post-transplant autoimmune hepatitis. Am J Transplant.

[314] Chatrath H, Allen L, Boyer TD (2014). Use of sirolimus in the treatment of refractory autoimmune hepatitis. Am J Med.

[315] Kanzler S, Gerken G, Dienes HP, zum Buschenfelde KHM, Lohse AW (1997). Cyclophosphamide as alternative immunosuppressive therapy for autoimmune hepatitis–report of three cases. Z Gastroenterol.

[316] Burak KW, Urbanski SJ, Swain MG (1998). Successful treatment of refractory type 1 autoimmune hepatitis with methotrexate. J Hepatol.

[317] van den Brand FF, van Nieuwkerk CMJ, Verwer BJ, de Boer YS, de Boer NKH, Mulder CJJ (2018). Biochemical efficacy of tioguanine in autoimmune hepatitis: a retrospective review of practice in the Netherlands. Aliment Pharmacol Ther.

[318] Burak KW, Swain MG, Santodomingo-Garzon T, Lee SS, Urbanski SJ, Aspinall AI (2013). Rituximab for the treatment of patients with autoimmune hepatitis who are refractory or intolerant to standard therapy. Can J Gastroenterol.

[319] Adam R, Karam V, Delvart V, O’Grady J, Mirza D, Klempnauer J (2012). Evolution of indications and results of liver transplantation in Europe. A report from the European liver transplant registry (ELTR). J Hepatol.

[320] Singal AK, Guturu P, Hmoud B, Kuo YF, Salameh H, Wiesner RH (2013). Evolving frequency and outcomes of liver transplantation based on etiology of liver disease. Transplantation.

[321] TJLT Society (2015). Liver transplantation in Japan- registry by the Japanese liver transplantation society- [in Japanese]. Ishoku.

[322] Ayata G, Gordon FD, Lewis WD, Pomfret E, Pomposelli JJ, Jenkins RL (2000). Liver transplantation for autoimmune hepatitis: a long-term pathologic study. Hepatology.

[323] Campsen J, Zimmerman MA, Trotter JF, Wachs M, Bak T, Steinberg T (2008). Liver transplantation for autoimmune hepatitis and the success of aggressive corticosteroid withdrawal. Liver Transpl.

[324] Duclos-Vallee JC, Sebagh M, Rifai K, Johanet C, Ballot E, Guettier C (2003). A 10 year follow up study of patients transplanted for autoimmune hepatitis: histological recurrence precedes clinical and biochemical recurrence. Gut.

[325] Gonzalez-Koch A, Czaja AJ, Carpenter HA, Roberts SK, Charlton MR, Porayko MK (2001). Recurrent autoimmune hepatitis after orthotopic liver transplantation. Liver Transpl.

[326] Krishnamoorthy TL, Miezynska-Kurtycz J, Hodson J, Gunson BK, Neuberger J, Milkiewicz P (2016). Longterm corticosteroid use after liver transplantation for autoimmune hepatitis is safe and associated with a lower incidence of recurrent disease. Liver Transpl.

[327] Milkiewicz P, Hubscher SG, Skiba G, Hathaway M, Elias E (1999). Recurrence of autoimmune hepatitis after liver transplantation. Transplantation.

[328] Molmenti EP, Netto GJ, Murray NG, Smith DM, Molmenti H, Crippin JS (2002). Incidence and recurrence of autoimmune/alloimmune hepatitis in liver transplant recipients. Liver Transpl.

[329] Montano-Loza AJ, Mason AL, Ma M, Bastiampillai RJ, Bain VG, Tandon P (2009). Risk factors for recurrence of autoimmune hepatitis after liver transplantation. Liver Transpl.

[330] Prados E, Cuervas-Mons V, de la Mata M, Fraga E, Rimola A, Prieto M (1998). Outcome of autoimmune hepatitis after liver transplantation. Transplantation.

[331] Ratziu V, Samuel D, Sebagh M, Farges O, Saliba F, Ichai P (1999). Long-term follow-up after liver transplantation for autoimmune hepatitis: evidence of recurrence of primary disease. J Hepatol.

[332] Reich DJ, Fiel I, Guarrera JV, Emre S, Guy SR, Schwartz ME (2000). Liver transplantation for autoimmune hepatitis. Hepatology.

[333] Montano-Loza AJ, Bhanji RA, Wasilenko S, Mason AL (2017). Systematic review: recurrent autoimmune liver diseases after liver transplantation. Aliment Pharmacol Ther.

[334] Visseren T, Murad SD (2017). Recurrence of primary sclerosing cholangitis, primary biliary cholangitis and auto-immune hepatitis after liver transplantation. Best Pract Res Clin Gastroenterol.

[335] Alvarez F, Berg P, Bianchi F, Bianchi L, Burroughs A, Cancado E (1999). International autoimmune hepatitis group report: review of criteria for diagnosis of autoimmune hepatitis. J Hepatol.

[336] Balan V, Ruppert K, Demetris AJ, Ledneva T, Duquesnoy RJ, Detre KM (2008). Long-term outcome of human leukocyte antigen mismatching in liver transplantation: results of the national institute of diabetes and digestive and kidney diseases liver transplantation database. Hepatology.

[337] Kerkar N, Vergani D (2018). De novo autoimmune hepatitis- is this different in adults compared to children?. J Autoimmun.

[338] Kerkar N, Yanni G (2016). ‘De novo’ and ‘recurrent’ autoimmune hepatitis after liver transplantation: a comprehensive review. J Autoimmun.

[339] Montano-Loza AJ, Vargas-Vorackova F, Ma M, Bain VG, Burak K, Kumar T (2012). Incidence and risk factors associated with de novo autoimmune hepatitis after liver transplantation. Liver Int.

[340] Gupta P, Hart J, Millis JM, Cronin D, Brady L (2001). De novo hepatitis with autoimmune antibodies and atypical histology: a rare cause of late graft dysfunction after pediatric liver transplantation. Transplantation.

[341] Pongpaibul A, Venick RS, McDiarmid SV, Lassman CR (2012). Histopathology of de novo autoimmune hepatitis. Liver Transpl.

[342] Czaja AJ (2012). Diagnosis, pathogenesis, and treatment of autoimmune hepatitis after liver transplantation. Dig Dis Sci.

[343] Lindor KD, Gershwin ME, Poupon R, Kaplan M, Bergasa NV, Heathcote EJ (2009). Primary biliary cirrhosis. Hepatology.

[344] Chazouilleres O, Wendum D, Serfaty L, Rosmorduc O, Poupon R (2006). Long term outcome and response to therapy of primary biliary cirrhosis-autoimmune hepatitis overlap syndrome. J Hepatol.

[345] Ozaslan E, Efe C, Heurgué-Berlot A, Kav T, Masi C, Purnak T (2014). Factors associated with response to therapy and outcome of patients with primary biliary cirrhosis with features of autoimmune hepatitis. Clin Gastroenterol Hepatol.

[346] Czaja AJ (2013). Diagnosis and management of the overlap syndromes of autoimmune hepatitis. Can J Gastroenterol.

[347] Floreani A, Rizzotto ER, Ferrara F, Carderi I, Caroli D, Blasone L (2005). Clinical course and outcome of autoimmune hepatitis/primary sclerosing cholangitis overlap syndrome. Am J Gastroenterol.

[348] Al-Chalabi T, Portmann BC, Bernal W, McFarlane IG, Heneghan MA (2008). Autoimmune hepatitis overlap syndromes: an evaluation of treatment response, long-term outcome and survival. Aliment Pharmacol Ther.

[349] Olsson R, Glaumann H, Almer S, Broome U, Lebrun B, Bergquist A (2009). High prevalence of small duct primary sclerosing cholangitis among patients with overlapping autoimmune hepatitis and primary sclerosing cholangitis. Eur J Intern Med.

[350] Castiella A, Zapata E, Lucena MI, Andrade RJ (2014). Drug-induced autoimmune liver disease: a diagnostic dilemma of an increasingly reported disease. World J Hepatol.

[351] Weber S, Benesic A, Rotter I, Gerbes AL (2019). Early ALT response to corticosteroid treatment distinguishes idiosyncratic drug-induced liver injury from autoimmune hepatitis. Liver Int.

[352] Björnsson E, Talwalkar J, Treeprasertsuk S, Kamath PS, Takahashi N, Sanderson S (2010). Drug-induced autoimmune hepatitis: clinical characteristics and prognosis. Hepatology.

[353] Terrault NA, Lok ASF, McMahon BJ, Chang KM, Hwang JP, Jonas MM (2018). Update on prevention, diagnosis, and treatment of chronic hepatitis B: AASLD 2018 hepatitis B guidance. Hepatology.

[354] EASL (2018). EASL recommendations on treatment of hepatitis C 2018. J Hepatol.

[355] De Luca-Johnson J, Wangensteen KJ, Hanson J, Krawitt E, Wilcox R (2016). Natural history of patients presenting with autoimmune hepatitis and coincident nonalcoholic fatty liver disease. Dig Dis Sci.

[356] Komura T, Ohta H, Seike T, Shimizu Y, Nakai R, Omura H (2018). The efficacy of corticosteroid therapy in a patient with non-alcoholic steatohepatitis overlapping autoimmune hepatitis. Intern Med.

[357] Wan DW, Marks K, Yantiss RK, Talal AH (2009). Autoimmune hepatitis in the HIV-infected patient: a therapeutic dilemma. AIDS Patient Care STDS.

[358] Tansel A, Katz LH, El-Serag HB, Thrift AP, Parepally M, Shakhatreh MH (2017). Incidence and determinants of hepatocellular carcinoma in autoimmune hepatitis: a systematic review and meta-analysis. Clin Gastroenterol Hepatol.

[359] Valean S, Acalovschi M, Dumitrascu DL, Ciobanu L, Nagy G, Chira R (2019). Hepatocellular carcinoma in patients with autoimmune hepatitis—a systematic review of the literature published between 1989–2016. Med Pharm Rep.

[360] Hino-Arinaga T, Ide T, Kuromatsu R, Miyajima I, Ogata K, Kuwahara R (2012). Risk factors for hepatocellular carcinoma in Japanese patients with autoimmune hepatitis type 1. J Gastroenterol.

[361] Migita K, Watanabe Y, Jiuchi Y, Nakamura Y, Saito A, Yagura M (2012). Hepatocellular carcinoma and survival in patients with autoimmune hepatitis (Japanese national hospital organization-autoimmune hepatitis prospective study). Liver Int.

[362] Montano-Loza AJ, Carpenter HA, Czaja AJ (2008). Predictive factors for hepatocellular carcinoma in type 1 autoimmune hepatitis. Am J Gastroenterol.

[363] Teufel A, Weinmann A, Centner C, Piendl A, Lohse AW, Galle PR (2009). Hepatocellular carcinoma in patients with autoimmune hepatitis. World J Gastroenterol.

[364] Wong RJ, Gish R, Frederick T, Bzowej N, Frenette C (2011). Development of hepatocellular carcinoma in autoimmune hepatitis patients: a case series. Dig Dis Sci.

[365] Borssen AD, Almer S, Prytz H, Wallerstedt S, Friis-Liby IL, Bergquist A (2015). Hepatocellular and extrahepatic cancer in patients with autoimmune hepatitis—a long-term follow-up study in 634 Swedish patients. Scand J Gastroenterol.

[366] Dakhoul L, Jones KR, Gawrieh S, Ghabril M, McShane C, Vuppalanchi R (2019). Older age and disease duration are highly associated with hepatocellular carcinoma in patients with autoimmune hepatitis. Dig Dis Sci.

[367] Ohira H, Abe K, Takahashi A, Zeniya M, Ichida T (2013). Clinical features of hepatocellular carcinoma in patients with autoimmune hepatitis in Japan. J Gastroenterol.

